# The *Giardia* ventrolateral flange is a lamellar membrane protrusion that supports attachment

**DOI:** 10.1371/journal.ppat.1010496

**Published:** 2022-04-28

**Authors:** William R. Hardin, Germain C. M. Alas, Nikita Taparia, Elizabeth B. Thomas, Melissa C. Steele-Ogus, Kelli L. Hvorecny, Aaron R. Halpern, Pavla Tůmová, Justin M. Kollman, Joshua C. Vaughan, Nathan J. Sniadecki, Alexander R. Paredez

**Affiliations:** 1 Department of Biology, University of Washington, Seattle, Washington, United States of America; 2 Department of Mechanical Engineering, University of Washington, Seattle, Washington, United States of America; 3 Department of Biochemistry, University of Washington, Seattle, Washington, United States of America; 4 Department of Chemistry, University of Washington, Seattle, Washington, United States of America; 5 Institute of Immunology and Microbiology, 1^st^ Faculty of Medicine, Charles University, Prague, Czech Republic; 6 Department of Physiology and Biophysics, University of Washington, Seattle, Washington, United States of America; 7 Bioengineering, University of Washington, Seattle, Washington, United States of America; 8 Lab Medicine & Pathology, University of Washington, Seattle, Washington, United States of America; 9 Center for Cardiovascular Biology, University of Washington, Seattle, Washington, United States of America; 10 Institute for Stem Cell and Regenerative Medicine, University of Washington, Seattle, Washington, United States of America; University of Virginia, UNITED STATES

## Abstract

Attachment to the intestinal epithelium is critical to the lifestyle of the ubiquitous parasite *Giardia lamblia*. The ventrolateral flange is a sheet-like membrane protrusion at the interface between parasites and attached surfaces. This structure has been implicated in attachment, but its role has been poorly defined. Here, we identified a novel actin associated protein with putative WH2-like actin binding domains we named Flangin. Flangin complexes with *Giardia* actin (*Gl*Actin) and is enriched in the ventrolateral flange making it a valuable marker for studying the flanges’ role in *Giardia* biology. Live imaging revealed that the flange grows to around 1 μm in width after cytokinesis, then remains uniform in size during interphase, grows in mitosis, and is resorbed during cytokinesis. A flangin truncation mutant stabilizes the flange and blocks cytokinesis, indicating that flange disassembly is necessary for rapid myosin-independent cytokinesis in *Giardia*. Rho family GTPases are important regulators of membrane protrusions and *Gl*Rac, the sole Rho family GTPase in *Giardia*, was localized to the flange. Knockdown of Flangin, *Gl*Actin, and *Gl*Rac result in flange formation defects. This indicates a conserved role for *Gl*Rac and *Gl*Actin in forming membrane protrusions, despite the absence of canonical actin binding proteins that link Rho GTPase signaling to lamellipodia formation. Flangin-depleted parasites had reduced surface contact and when challenged with fluid shear force in flow chambers they had a reduced ability to remain attached, confirming a role for the flange in attachment. This secondary attachment mechanism complements the microtubule based adhesive ventral disc, a feature that may be particularly important during mitosis when the parental ventral disc disassembles in preparation for cytokinesis. This work supports the emerging view that *Giardia’s* unconventional actin cytoskeleton has an important role in supporting parasite attachment.

## Author summary

The ventrolateral flange is a lamella found at the host-parasite interface that has long been thought to be involved in parasite attachment. Researchers have not identified proteins responsible for building the flange, precluding manipulation of the flange to determine its role in *Giardia* biology. We identified Flangin, a novel actin associated protein that localizes to the flange, implicating *Gl*Actin in flange formation. We demonstrate that: 1) Flangin, *Gl*Actin, and *Gl*Rac are required for flange formation, 2) the flange grows in width during mitosis when the ventral disc is being broken down and then it is resorbed during cytokinesis when cells detach to swim apart, and 3) the flange augments attachment, which is critical to parasitism. The presence of redundant attachment mechanisms underscores the importance of attachment to the lifestyle of this ubiquitous parasite.

## Introduction

*Giardia lamblia* (synonymous with *G*. *intestinalis* and *G*. *duodenalis)* is a common waterborne pathogen that infects more than 200 million people each year [1,2]. Infection begins with ingestion of a cysts which gives rise to two trophozoites after passage through the acidic environment of the stomach. Trophozoites then colonize the intestine as extra cellular parasites that use their cytoskeleton to attach to the intestinal epithelium [3]. The prominent microtubule based ventral adhesive disc plays a central role in supporting parasitic attachment [4]. Recently we used proteomics to identify actin binding proteins in *Giardia* and found several that localized to portions of the ventral disc which indicates that the actin cytoskeleton also plays a role in parasitic attachment [5,6].

The actin cytoskeleton is essential to maintaining cell shape and forms one of the central organizing scaffolds of eukaryotic cells. Most of our understanding of actin regulation and function comes from studies performed in a limited number of model eukaryotes [7,8]. *Giardia lamblia* belongs to a potentially early diverging eukaryotic lineage, lacks all canonical actin-binding proteins used to regulate actin dynamics, including myosin, formin, cofilin, and the Arp2/3 complex, which is consistent with *Giardia* having the most divergent eukaryotic actin identified to date [9–13]. These unique attributes make *Giardia* a valuable model for studying novel mechanisms in regulating the assembly of actin microfilaments [3].

Despite the lack of canonical actin-binding proteins, *Giardia* has a dynamic actin cytoskeleton that is required for conserved roles including membrane trafficking, cell shape, cell polarity, and cytokinesis [14,15]. Dynamic reorganization of *Giardia lamblia* actin (*Gl*Actin) during the cell cycle suggests the presence of actin regulatory proteins. A previous effort at biochemically isolating actin binding proteins from *Giardia* identified several proteins that associate with *Gl*Actin [16]. However, these proteins likely associate with monomeric actin, given the biochemical conditions used to isolate these proteins and the lack of filamentous localization patterns observed by immunofluorescence microscopy [16,17]. Therefore, the previously identified proteins are unlikely to account for the full complement of actin binding proteins in *Giardia*.

Many actin binding proteins have a small helical domain known as the Wasp Homology 2 (WH2) domain. This versatile domain is found in proteins that account for the most basic forms of actin regulation such as monomer sequestration, nucleation, capping, and depolymerization [18,19]. WH2 sequences are short 15–20 amino acid helices that bind to the hydrophobic cleft at the barbed (+) end of actin monomers, which avoids most cross strand interfaces of actin filaments permitting their use for regulatory functions with both monomeric and filamentous actin [20]. *Giardia* actin is highly divergent with 58% average identity to conventional actins (yeast to human), yet the hydrophobic cleft is well conserved, suggesting that WH2 or WH2-like domains could regulate actin in *Giardia* (S1 Fig).

Here we used a bioinformatics approach to identify putative WH2-like domain containing proteins and identified Flangin, which we find localizes to *Giardia’s* lamellipodium-like ventrolateral flange. The flange is a thin flexible membrane protrusion encircling the ventro-lateral periphery of trophozoites, which is the life stage that colonizes the intestine. Although outwardly similar to lamellipodia, the flange is not known to have any role in motility and *Giardia* lacks the Arp2/3 complex which plays a central role in lamellipodia formation [21–23]. Based upon morphology and location, it has been suggested that the flange plays a role in attachment [24–26]. Live cell imaging has revealed that the flange is the first part of the cell to engage with surfaces during attachment [27]. However, proximity to attached surfaces does not necessarily indicate a functional role in attachment. The only experimental support for the ventrolateral flange participating in attachment comes from a study where the ventral disc was mechanically impeded from establishing suction with a microfabricated surface of posts; a small subset of cells still managed to attach with what were interpreted as adhesive contacts between the flange and the microfabricated surface [28]. Molecular components of the flange were unknown, preventing further testing of the flange’s role in *Giardia* attachment.

Our identification of a flange component presented the opportunity to explore the role of the flange in attachment as well as determine if the ventrolateral flange formation has any similarities to lamellipodia. We demonstrate that Flangin, *Gl*Actin, and *Giardia’s* sole Rho family GTPase, *Giardia lamblia* Rac (*Gl*Rac), all have roles in flange formation. The ability to perturb flange formation allowed us to separate ventral disc engagement with the attached surface from typical flange and cell body contacts observed during the progression of attachment. Attachment assays performed under fluid flow confirmed that the ventrolateral flange augments parasite attachment. This finding is important because most studies aimed at understanding *Giardia* attachment have focused on the suction-cup like microtubule based adhesive ventral disc [4,13]. Our findings indicate that the ventral disc is only part of the attachment mechanism. Roles for *Gl*Actin in attachment have been controversial due to conflicting results with actin inhibitors that are not efficacious against *Giardia’s* highly divergent actin [13,14]. The importance of *Gl*Actin in flange formation and the role of the flange in attachment highlights the importance of the actin cytoskeleton to *Giardia* attachment.

## Results

### Identification of Flangin

To identify putative actin-binding proteins, we searched the *Giardia* genome for WH2 domain containing proteins using PHI blast (see **Materials and Methods**). Our search resulted in the identification of 216 proteins that contain one or more putative WH2-like domains (S1 Table). Our search parameters were low stringency; while we likely have a high false positive rate, the comprehensive list is a valuable starting point for this and future studies. Since *Giardia* lacks the Arp2/3 complex and formin actin nucleators, we were particularly interested in proteins with multiple WH2-like domains. Such proteins could potentially have a role in actin nucleation, which is critical to cytoskeletal regulation [20,29]. Our search yielded eight proteins with multiple predicted WH2 domains. One protein, GL50803_7031, hereafter referred to as Flangin, was particularly intriguing because in addition to three putative WH2-like domains, this protein also possesses a Bro1 domain (Interpro E = 2^−13^) at the N-terminus that is implicated in F-actin binding (S2 Fig) [30,31].

**Fig 1:**
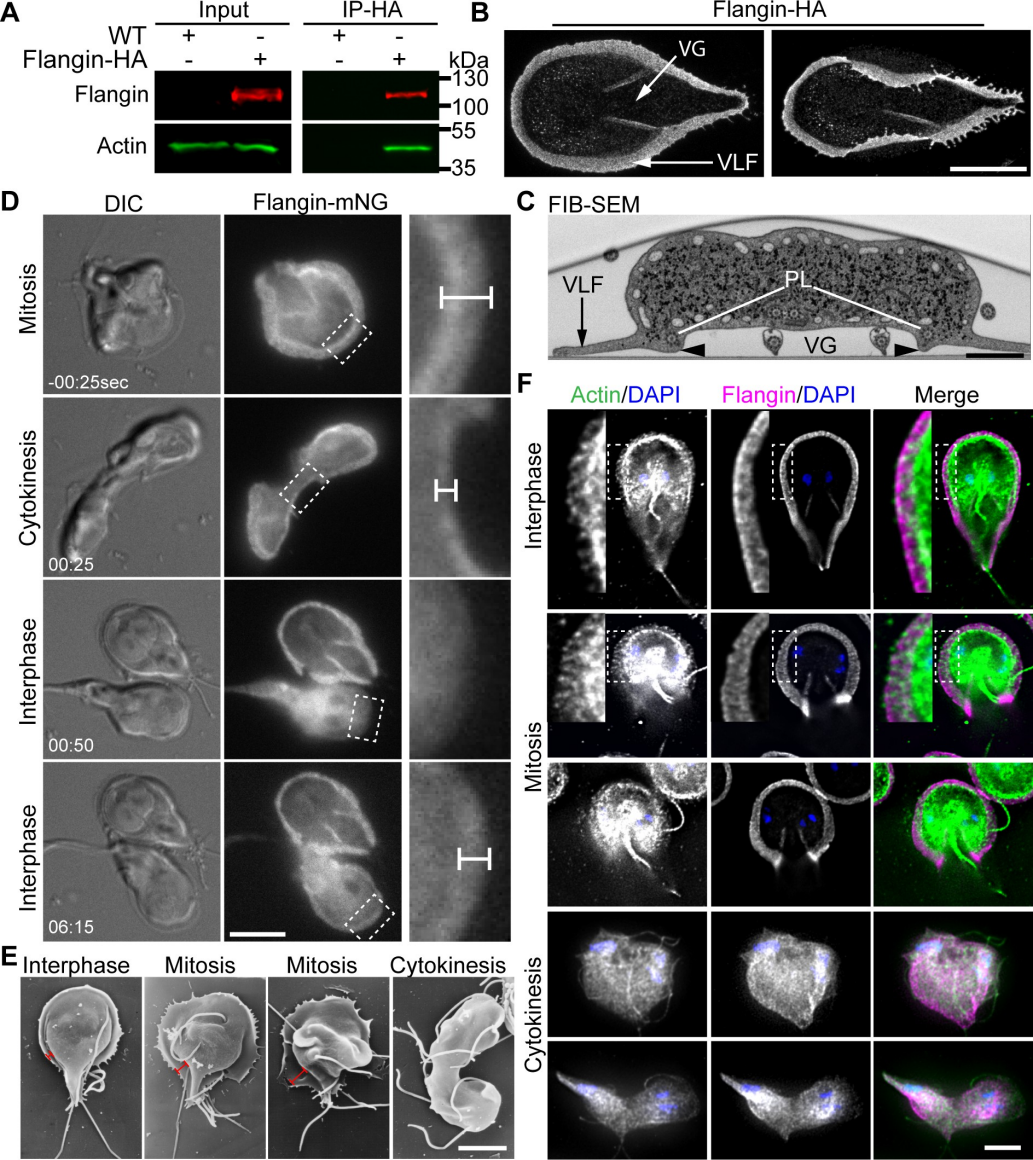
*Giardia* possesses a lamellipodium-like structure that contains *Gl*Actin and Flangin. (A) Immunoprecipitation of Flangin-HA from cell extracts, followed by anti-*Gl*Actin Western blotting demonstrates Flangin associates with *Gl*Actin. (B) Maximum projections of expanded trophozoites imaged with confocal fluorescence microscopy. Flangin-HA marks the ventrolateral flange (VLF) and membrane protrusions associated with the cytoplasmic portions of the posterolateral flagella axonemes that bound the ventral grove (VG). The left image shows that the flange can completely encircle the base of the cell and the image on the right shows that the flange is thin and flexible. (C) Transverse section of wild-type *Giardia* (posterior to the ventral flagella exit point) as viewed with Focused Ion Beam-Scanning Electron Microscopy (FIB-SEM). Arrowhead marks the membrane protrusion associated with Flangin and the cytoplasmic axonemes of the posterolateral (PL) axonemes that bound the ventral groove. (D) Live cell imaging of Flangin-mNG during cytokinesis. Compared with interphase cells the flange is wider in mitosis and was resorbed during cytokinesis. Upon cytokinesis, the flange disassembled and Flangin-mNG translocated to the cytoplasm. Daughter cells lack a flange, but assembly was initiated almost immediately after division. Flangin-mNG was recruited back to the flange as it reformed (see S1 Movie). Magnified views of the boxed area show Flangin is at the leading edge of the flange. Dimension bars in C and D point out flange width. (E) Scanning electron microscopy shows an expanded flange in mitosis and the lack of a flange during cytokinesis. (F) Immunofluorescence localization of *Gl*Actin (green), Flangin-HA (magenta), and DNA (blue), throughout the cell cycle (see S3 Fig for tubulin staining). *Gl*Actin and Flangin accumulate in the flange during interphase and mitosis. The insets show a magnified view of *Gl*Actin and Flangin in the flange. During cytokinesis flange localized Flangin and *Gl*Actin translocated to cytoplasm as the flange is disassembled. Note that the interphase and mitotic cells are partial projections optimized to show *Gl*Actin localization, while the entire Z-stack was projected for the cells in cytokinesis to show that the flange has been resorbed. Scale bars = 5 μm except (C) = 1 μm.

Due to the presence of putative actin binding domains, we proceeded to test for interaction with *Gl*Actin. Flangin was endogenously tagged with a triple hemagglutinin epitope (Flangin-3HA) using single site recombination [32]. Prior to making cell extracts the cells were crosslinked with membrane permeable DSP to preserve protein-protein interactions. After pulldown with Anti-HA beads the presence of endogenous *Gl*Actin was detected using a custom anti-*Gl*Actin antibody [14]. Western blotting detected *Gl*Actin in the Flangin-HA pulldown but not in the wild-type negative control indicating specific molecular association of Flangin and *Gl*Actin (Fig 1A).

Flangin-3HA was then localized with expansion microscopy, a super-resolution microscopy method which provided down to 70 nm spatial resolution, or roughly four times the resolution of conventional confocal microscopy [33,34]. We found that Flangin-3HA localized to the ventrolateral flange (Fig 1B). In addition to localization at the ventrolateral flange, Flangin was found along the boundaries of the ventral groove (Fig 1B and 1C). Together the ventral groove and finned ventral flagella are thought to function as a pump that pushes fluid from underneath cells to generate a negative pressure differential supporting parasite attachment [35–37].

### Ventrolateral flange morphology is cell cycle regulated

To follow Flangin dynamics over the cell cycle, we tagged Flangin with mNeonGreen (Flangin-mNG) and performed live cell imaging. Relative to interphase, the flange width increased by 69% in mitosis (0.93 ± 0.23 μm to 1.57 ± 0.44 μm n = 18). Ventrolateral flange expansion began in anaphase so that the expanded flange was in place when the ventral disc began disassembly at the anaphase/telophase transition (S3A Fig [15,38,39]). At the onset of cytokinesis, Flangin re-localized from the flange to the cytoplasm as the flange was resorbed (Fig 1D and S1 Movie). Disassembly of the flange could be important for promoting detachment, which precedes daughter cells swimming in opposite directions to drive furrow progression and abscission [15,39,40]. Within 60 seconds of completing cytokinesis, daughter cells initiated flange protrusion at their anterior (S2 Movie). After flange assembly was initiated at the anterior, flange protrusion propagated along the cell body with the posterior region of the flange being the last to complete growth. Daughter cells noticeably elongated and restored their typical shape during posterior flange expansion (S2 Movie). On average, the flange reached a maximum width of 0.97 ± 0.079 μm after 155 ± 32 seconds with a protrusion rate of 0.39 ± 0.1 μm/min (n = 20), which is near the protrusion rate of lamellipodia [41,42]. Unlike lamellipodia, which continuously grow and shrink to drive cell motility, flanges grew without apparent shrinking or retracting before reaching the final interphase width (S1 and S2 Movies). Consistent with previous observations [39], flange width in scanning electron microscopy micrographs of wild-type trophozoites qualitatively correlated with light microscopy observations (Fig 1E).

### Actin is a component of the ventrolateral flange

Given the biochemical association of Flangin with *Gl*Actin, we assessed *Gl*Actin presence in the flange. We imaged actin in fixed cells using a *Gl*Actin specific antibody because efforts to fluorescently tag *Gl*Actin or use conventional live actin reporters such as LifeAct or SiR-Actin have been unsuccessful [14]. Both filamentous *Gl*Actin and Flangin-3HA, were found throughout the flange in interphase and mitotic trophozoites (Fig 1F). Additionally, Flangin was observed in association with the ends of *Gl*Actin filaments in the cell body (S3B Fig). Together, our results indicate that Flangin and *Gl*Actin are associated and are components of the flange.

### Flangin and *Gl*Actin are necessary for ventrolateral flange assembly

**Fig 2:**
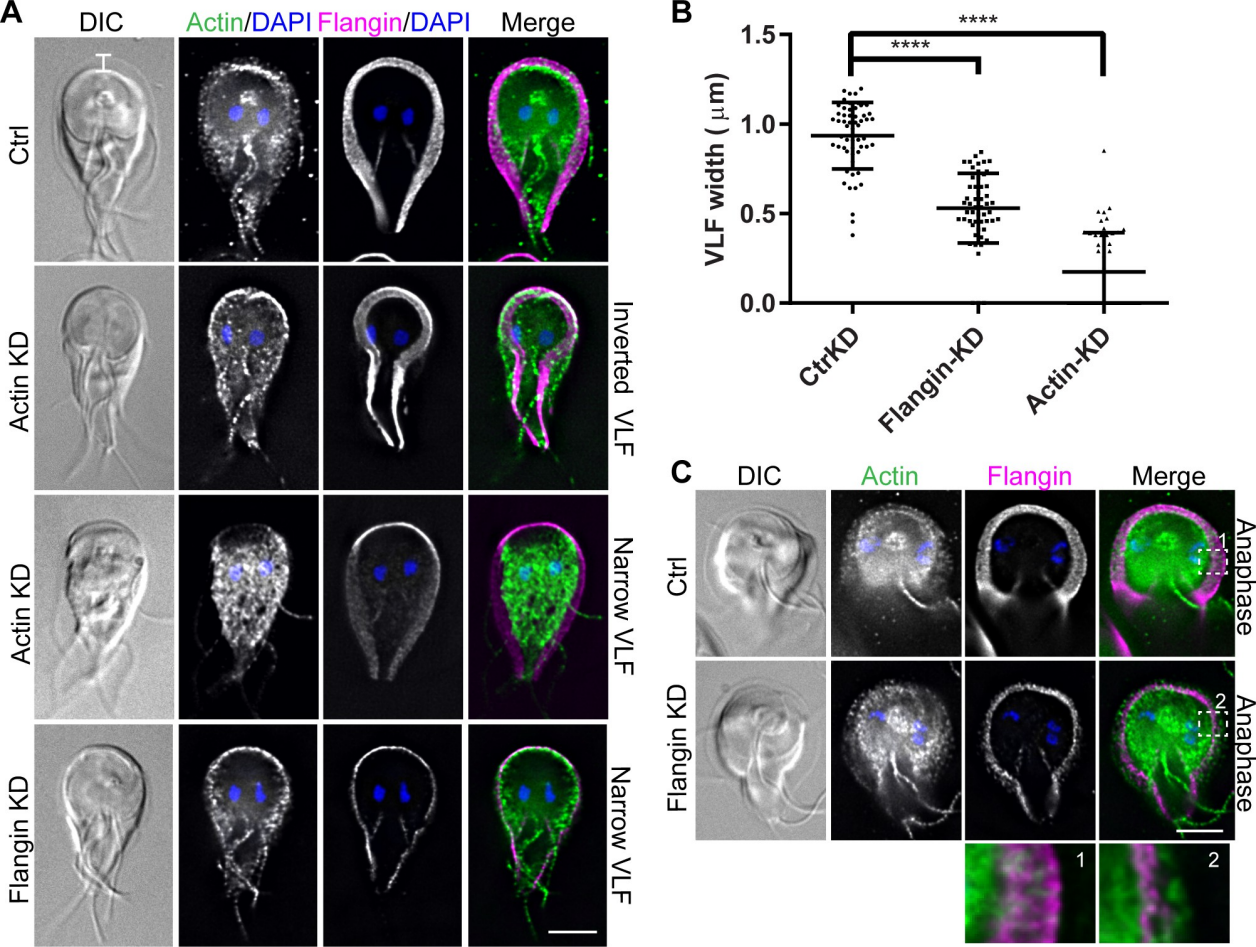
Flangin and *Gl*Actin are necessary for flange assembly. (A) Trophozoites were stained for *Gl*Actin (green), Flangin-HA (magenta), and DNA (blue). Note that images are scaled to display the remaining protein and are not intended to show differences in protein levels. *Gl*Actin knockdown (KD) resulted in inverted and collapsed flange morphology. Flangin-HA KD similarly resulted in a thin flange phenotype. (B) Quantification of flange width when measured at the cell anterior from three independent experiments; control (n = 57), Flangin-HA (n = 54), and actin (n = 51). Statistical significance was evaluated for Flangin-KD and Actin-KD respectively, t test. ****, P<0.0001. (C) Mitotic Flangin depleted cells were capable of extending their flange beyond the leading edge of Flangin. The insets show actin beyond the leading edge of Flangin-HA in Flangin-HA KD cells during mitotic flange extension. Scale bars = 5 μm.

To reveal if Flangin and *Gl*Actin are required for flange formation, we depleted these proteins and measured the impact on the width of the flange. Translation-blocking antisense morpholinos were used to deplete *Gl*Actin, Flangin, or human beta-globin as a negative control. Quantitative western blotting revealed around 60% depletion of *Gl*Actin at the population level, 24 hours after morpholino treatment (S4 Fig). Knocking down actin, resulted in either an inverted or narrow flange phenotype compared to the negative morpholino control (Fig 2A). Although actin depletion can cause severe cellular disorganization [14], flange defects were observed in cells that retained typical organization as indicated by nuclear and flagella positioning (Fig 2A). For cells with the inverted phenotype, the flange did not visible extend from the cell body when observed by DIC microscopy, but Flangin was found to be localized around the base of the cell (23.2% of cells had the inverted phenotype, n = 73). Overall, *Gl*Actin depletion significantly decreased ventrolateral flange width (Fig 2A and 2B). An antisense-translation blocking morpholino targeting Flangin achieved around 70% knockdown at 24h (S4B Fig). Flangin-depleted cells had a narrow flange phenotype when compared to the control morpholino treated cells (Fig 2A and 2B). However, during mitosis when the flange of control cells grows in width, we were able to find Flangin-depleted cells with expanded flanges where *Gl*Actin but not Flangin was found at the leading edge of the flange (Fig 2C). These results are consistent with a role for *Gl*Actin in driving membrane protrusion and a role for Flangin in stabilizing the ventrolateral flange.

### Flangin is a stable component of the interphase ventrolateral flange

**Fig 3:**
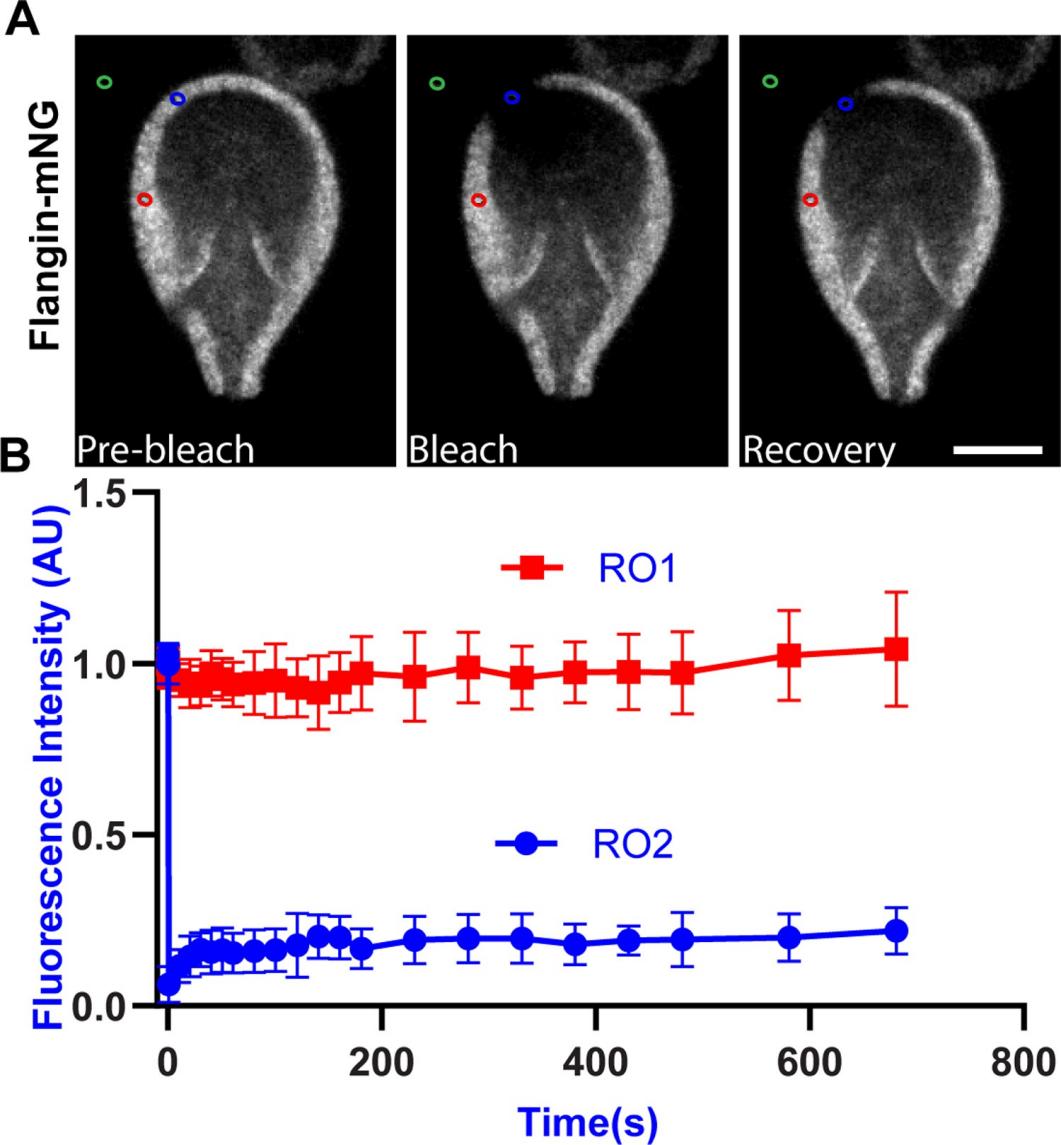
Flangin is part of a stable structure. (A) Fluorescence Recovery after Photobleaching (FRAP) was performed on the flange of Flangin-mNG cells, Blue circle bleached ROI 01, Red circle non-bleached ROI 02, and Green circle background ROI 03. (B) ROI 01 showed minimal post-bleaching recovery after 12min, n = 4 independent experiments. A lack of fluorescence recovery was also observed in 29 additional FRAP experiments of various lengths and regions of the cell including the small membrane protrusion associated with the posterolateral axonemes. Scale bar = 5 μm. See S3 Movie for confirmation of viability.

In contrast to lamellipodia, which are continuously remodeled to drive cell motility, ventrolateral flange size under our imaging conditions is static after initial re-growth in interphase (S2 Movie). We postulate that the flange could maintain a fixed width either through a balance of disassembly and assembly processes or the use of a static scaffold. Without a live *Gl*Actin marker, it is not currently possible to assess actin dynamics. To probe whether Flangin is a dynamic or stable component of the assembled flange, we assessed Flangin dynamics with fluorescence recovery after photobleaching (FRAP). FRAP of Flangin-mNG revealed minimal recovery in experiments with recovery times varying from three to twelve minutes (n = 35 cells, Fig 3A and 3B and S3 Movie). We conclude that Flangin is a stable structural component of the ventrolateral flange.

We also tested whether the N-terminal Bro1 domain (amino acids 1–362) or C-terminal WH2 region (amino acids 301–995) of Flangin are sufficient to localize the protein to the flange in a wildtype background. The N-terminal Bro1 domain is sufficient to recruit Flangin to the ventrolateral flange whereas the C-terminal fragment was distributed through the cell body with some enrichment at the cell cortex but not the ventrolateral flange or the boundary of the ventral groove (S6 Fig). Interestingly, Bro1 domains are membrane binding proteins that could have a role in inducing membrane curvature or sensing curvature similar to BAR domains, which are not found in *Giardia* [43–45]. The Bro1 domain is sufficient for recruitment to the ventrolateral flange, but whether this is mediated by interaction with specific lipids, interacting proteins, or the unique membrane curvature of the flange remains to be determined.

### Ventrolateral flange disassembly is required for cytokinesis

**Fig 4:**
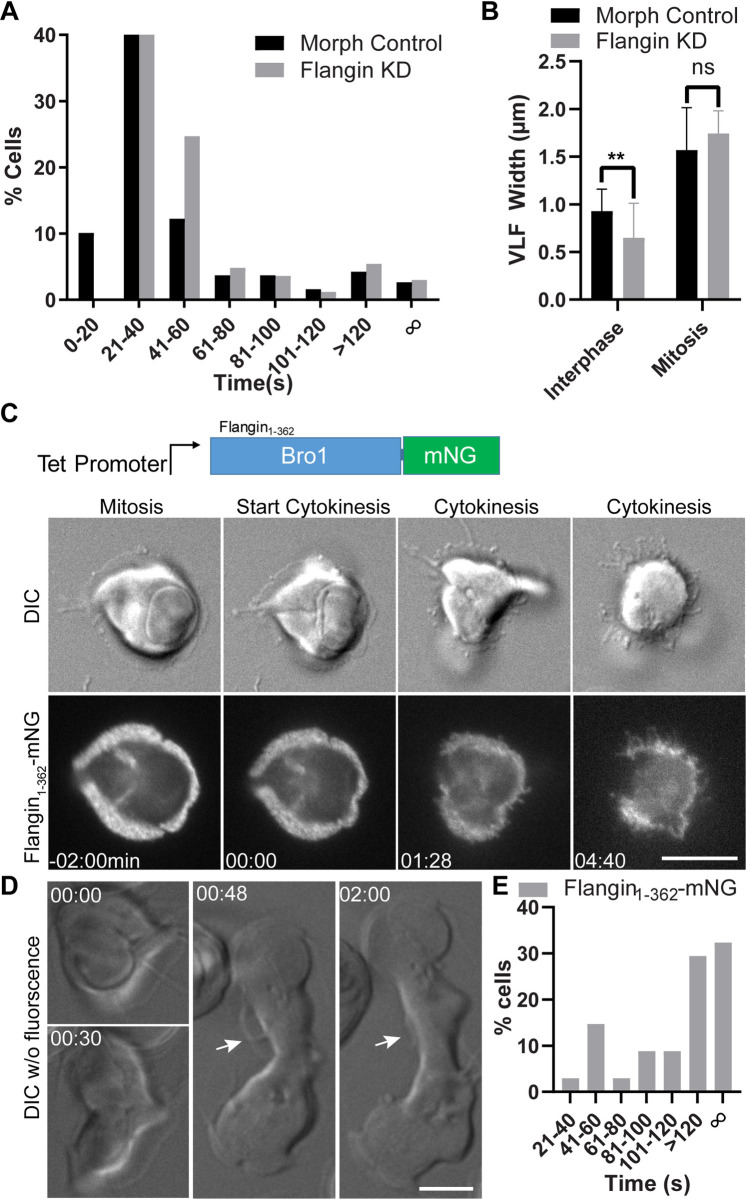
Flange breakdown is necessary for cleavage furrow progression. (A) Histogram showing the timing of cytokinesis for morpholino control (black) and Flangin KD cells (gray). See S4 and S5 Movies. Cytokinesis median time: control 31 sec (n = 188) and Flangin KD 35 sec (n = 166). Due to sampling frequency (timing between image stacks) the difference in median values in this experiment is not meaningful, similar to our previous study, more than 90% of the cells in both groups complete cytokinesis by 120 seconds indicating that there is no cytokinesis defect. (B) Flangin KD cells grow their flange before cytokinesis similar to control cells (See S5 Movie). (C) A tet inducible N-terminal truncation of Flangin interferes with flange retraction during cytokinesis. This blocks furrow progression and the ability of the parasite to lift off the cover glass (Compare S1 and S6 Movies). (D) DIC imaging of the same cell line without fluorescence. This is an example of a moderate defect where the flange persists and cytokinesis took 8 minutes to complete (see S7 Movie). White arrow points to regions of the flange that should have been resorbed by this point in cytokinesis. (E) Cytokinesis timing for Flangin_1-362_-mNG filmed with DIC alone (n = 34), only 38.3% of cells completed cytokinesis within 120 seconds. Scale bar = 5 μm.

Our observation that the flange expanded in mitosis and was resorbed during cytokinesis raised the possibility that flange membrane or other components could be used to support *Giardia’s* rapid cytokinesis. To test for a role in cytokinesis, we filmed Flangin-depleted cells using four-dimensional (4D) DIC microscopy (Fig 4A and S4 and S5 Movies). We did not observe delays or defects in cytokinesis when Flangin was depleted. Our previous analysis of *Giardia* cytokinesis found that 89% of wild type cells complete cytokinesis within two minutes with a median time of 50 seconds [15]. Here, more than 90% of morpholino control or Flangin-depleted cells completed cytokinesis in under two minutes. The median times for cytokinesis was 31 seconds for the morpholino control (n = 188, StdDev = 146 seconds) and 35 seconds for the Flangin-depleted cells (n = 166, StdDev = 162 seconds). The small difference in timing represents a sampling issue (number of optical sections in each image stack and interval between image stacks changes sampling) and is not biologically meaningful.

Flangin-depleted cells with narrow flange widths were able to expand their flanges in mitosis (S5 Movie). We found that Flangin-depleted cells increased their flange width during mitosis by about three-fold (Fig 4B). There was no significant difference in the maximum flange width immediately before cytokinesis for Flangin-depleted cells (1.76±0.25 μm) and morpholino control cells (1.57 ± 0.44). Thus, Flangin-depleted cells effectively establish an expanded flange before cytokinesis, preventing us from making any conclusions about the role of the flange in supporting cytokinesis.

While Flangin depletion did not impair cytokinesis, the tetracycline inducible N-terminal Bro1 fragment fused to mNeonGreen (Flangin_1-362_-mNG) had dominant negative function that impaired cytokinesis (S5 Fig). Live imaging of Flangin_1-362_-mNG revealed that the truncation protein impairs cytokinesis by stabilizing the ventrolateral flange. We observed two types of cytokinesis defects: complete failure or partial furrow progression (n = 21) (Fig 4C and S6 Movie). The example in Fig 4C is the more severe phenotype of complete failure to divide where stabilized regions of the flange result in filipodia like projections seen in six cells. The other 15 cells had some furrow progression, where portions of the flange did not disassemble, resulting in failed cytokinesis where one emerging daughter was smaller perhaps due to competition for plasma membrane.

When filming fluorescently tagged proteins, we sometimes observed mitotic cells that stalled in cytokinesis or never initiated cytokinesis after completing mitosis, likely a result of oxidative stress [46]. Therefore, we examined this cell line using long-term DIC imaging to avoid oxidative stress. Under DIC imaging, the cytokinesis failure rate of wild type and control cells is between 2% to 3% [15,47]. When Flangin_1-362_-mNG was filmed with 4D DIC imaging, 53% of cells took more than 2 minutes to divide and 32% failed to complete cytokinesis (n = 34, Fig 4D and 4E and S7 Movie), confirming that the N-terminal fragment interferes with the dynamics of the flange and cytokinesis. Taken together, our imaging results illustrate a requirement for disassembly of the ventrolateral flange for cytokinesis.

### *Gl*Rac has a conserved role in mediating actin-based membrane protrusions

**Fig 5:**
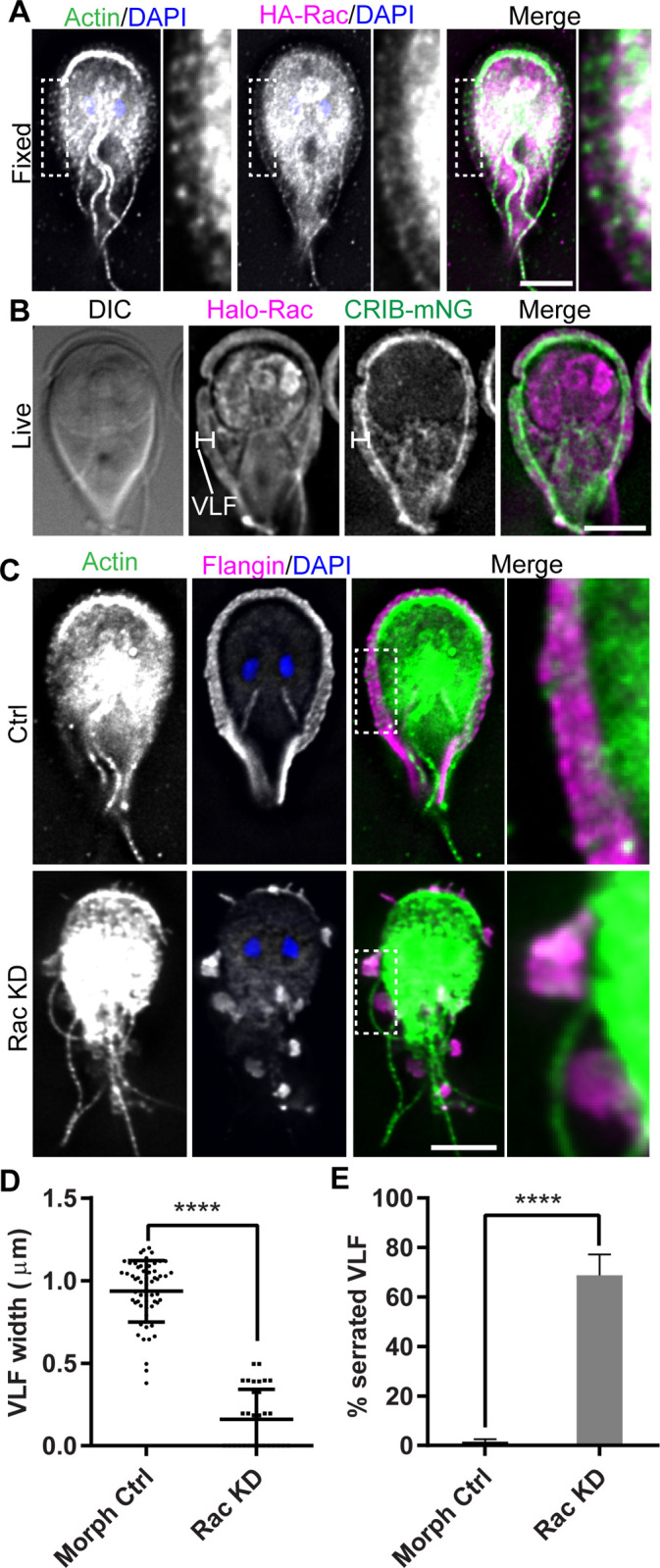
*Gl*Rac signaling is required for flange assembly. (A) Immunofluorescence localization of *Gl*Actin (green), HA-Rac (magenta), and DNA (blue). *Gl*Actin and *Gl*Rac localize to the flange. (B) Live cell imaging of Halo-Rac (magenta) and CRIB-mNG (green) indicate that *Gl*Rac is actively signaling in the flange. (C) *Gl*Rac KD resulted in a serrated flange phenotype not observed in control cells. *Gl*Actin and Flangin localize to the remaining sporadic flange protrusions. (D) Quantification of flange width measured at cell anterior from three independent experiments: control (n = 57) and *Gl*Rac KD (n = 32). Statistical significance was evaluated using the t-test. ****, P<0.0001. (E) Quantification of serrated cells. Cells with three or more flange gaps were defined as serrated; control (n = 596) and *Gl*Rac KD (n = 618). Statistical significance was evaluated using the t-test. ****, P< 0.0001. Scale bars = 5 μm.

Rho family GTPases are important regulators of actin-based membrane protrusions in model eukaryotes [48]. We questioned whether *Gl*Rac (GL50803_8496), *Giardia*’s sole Rho family GTPase, might also regulate flange formation. N-terminally HA-Tagged *Gl*Rac localized to the flange in a striated pattern with enrichment at the plasma membrane (Fig 5A). Live imaging of Halo-*Gl*Rac, which avoids the use of detergents necessary for immunofluorescence localization but known to disrupt Rho GTPase localization [49], indicated a more uniform distribution of *Gl*Rac in the flange.

Since Rho GTPase activity is regulated by nucleotide state, we sought to determine if *Gl*Rac is actively signaling at the flange. CRIB/PBD domains are specifically recruited to active GTP-loaded Rho GTPases and are commonly used as the basis for Rho GTPase signaling biosensors [50,51]. P21 activated Kinase (PAK, GL50803_2796) contains the only CRIB/PBD domain in the *Giardia* genome. We developed a CRIB based *Gl*Rac biosensor by fusing the CRIB/PBD domain of *Giardia’s* PAK kinase with mNG to create CRIB-mNG [52]. We found that CRIB-mNG was robustly recruited to the base of the ventrolateral flange, suggesting a role for active GTP-loaded *Gl*Rac in regulating flange assembly from this position (Fig 5B).

To determine if *Gl*Rac signaling is necessary for flange formation, we depleted *Gl*Rac with translation blocking morpholinos. *Gl*Rac was depleted by approximately 70% as compared to the morpholino control 24 hours after morpholino treatment (S4C Fig). Flange morphology was disrupted by *Gl*Rac knockdown resulting in flange morphologies that were patchy or serrated in profile (Fig 5C). *Gl*Actin and Flangin were absent from gaps in the flange but were found in all remaining flange protrusions. Quantification of the *Gl*Rac knockdown phenotypes indicated a significant decrease in the anterior flange width and a significant number of cells with a serrated phenotype (flange with 3 or more gaps) that was not observed in control cells (Fig 5D and 5E). We interpret these results to indicate that Flangin and *Gl*Actin recruitment is downstream of *Gl*Rac signaling and that the role of Rho GTPases regulating membrane protrusions is broadly conserved.

### The ventrolateral flange contributes to attachment

Early studies of *Giardia* attachment found that cells could attach under conditions where the ventral disk could not be employed [28]. Likewise, disruption of ventral disc organization is not sufficient to completely disrupt attachment [53,54]. Defects are only observed after challenge assays, which is consistent with redundant means of attachment. We sought to determine if disrupting the ventrolateral flange would impair attachment.

First, a basic attachment assay was performed where the proportion of trophozoites that could adhere to the side of culture tubes was determined. We depleted *Gl*Actin, *Gl*Rac, and Flangin to test their role in attachment. We observed fewer *Gl*Rac- and *Gl*Actin-knockdown cells attached to the culture tubes compared to the control (S7 Fig). The reduced attachment of *Gl*Rac- and *Gl*Actin-depleted cells likely resulted from gross disorganization of the cytoskeleton [14] which can disruption both the flange and ventral disc [6]. Due to multiple roles for each of these proteins in cellular organization specific conclusions about the role of the ventrolateral flange in attachment cannot be made with this assay. However, knockdown of Flangin did not disrupt attachment (S7 Fig).

**Fig 6:**
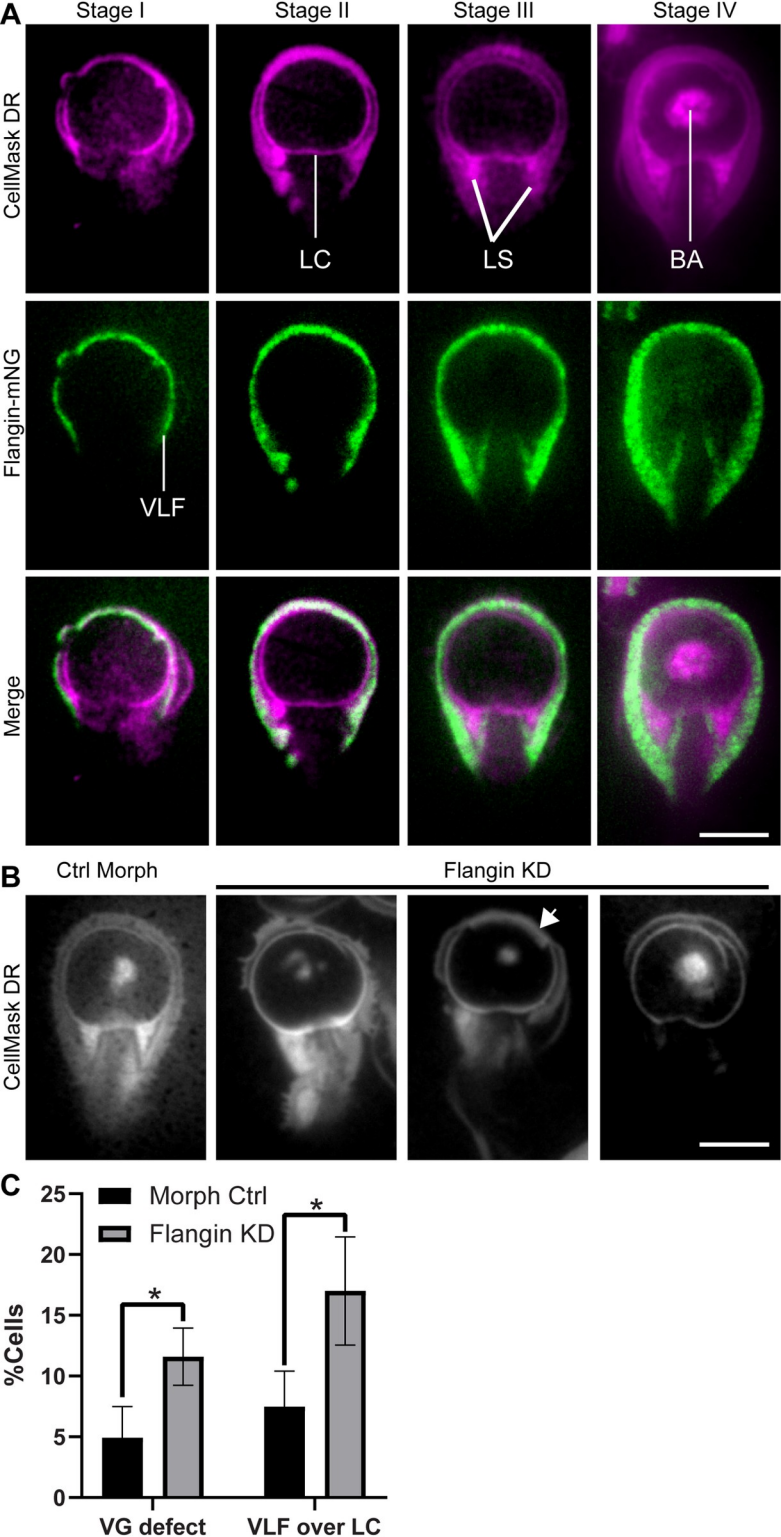
The flange makes intimate contact with attached surfaces and is required for normal ventral groove morphology. (A) Two color total internal reflection (TIRF) microscopy. Membranes are stained with CellMask Deep Red (magenta) and the flange is labeled with Flangin-mNG. As attachment progresses the flange becomes intimately associated with the substrate surface. (B) Knockdown of Flangin with translation blocking morpholinos uncouples the normal progression of attachment. Note that the ventral disc and bare area continues to make contact with the surface, but lateral shields (LS) do not make their typical contact with the attachment surface. This results in disruption of ventral groove channel important for fluid flows under the cell. Arrowhead points to an example of the flange being improperly positioned under the lateral crest (LC) where it may interfere with normal attachment. (C). Quantification of cells with ventral groove (VG) defects and cells with the flange positioned under the LC. Data are from three independent replicates including at least 100 cells each.

Since Flangin depletion disrupts flange maintenance but not overall cytoskeletal organization, Flangin knockdown permits direct assessment of the role of the ventrolateral flange in attachment. We analyzed cell surface contacts using total internal reflection (TIRF) microscopy, which limits signal detection to within 60–200 nm of the cover glass. CellMask Deep Red plasma membrane stain was used to visualize all surface contacts including engagement of the ventral disc, while Flangin-mNG allowed us to specifically observe the ventrolateral flange (Fig 6A). In agreement with previous studies [27,53], the control cells attached in a stepwise progression of attachment. In Stage I of attachment cells skim the surface and the primary contact with the surface is the flange. In Stage II the lateral crest of the ventral disc (border of disc) forms a complete seal with the surface. In Stage III the cells pull down tighter to the surface and the lateral shield, which forms the base of the ventral groove arch, comes into intimate contact with the cover glass. Finally in Stage IV the bare area of the ventral disc touches down. As these stages progress the flange becomes more intimately associated with the attached surface and the full extent of the flange becomes visible because the entire structure is encompassed within the TIRF evanescent field. Live imaging revealed that the flange can unfurl into position as attachment progresses, suggesting that its conformation may be actively regulated (S8 Movie).

Depletion of Flangin disrupted ventrolateral flange-based surface contacts (Fig 6B). Interestingly, disruption of the ventrolateral flange and ventral groove did not disrupt the ability of the ventral disc to adopt the fully engaged conformation as indicated by complete lateral crest seals and bare area contact (Fig 6B and 6C). This suggests that the ventral disc and ventrolateral flange have separable roles in mediating engagement with attached surfaces.

**Fig 7:**
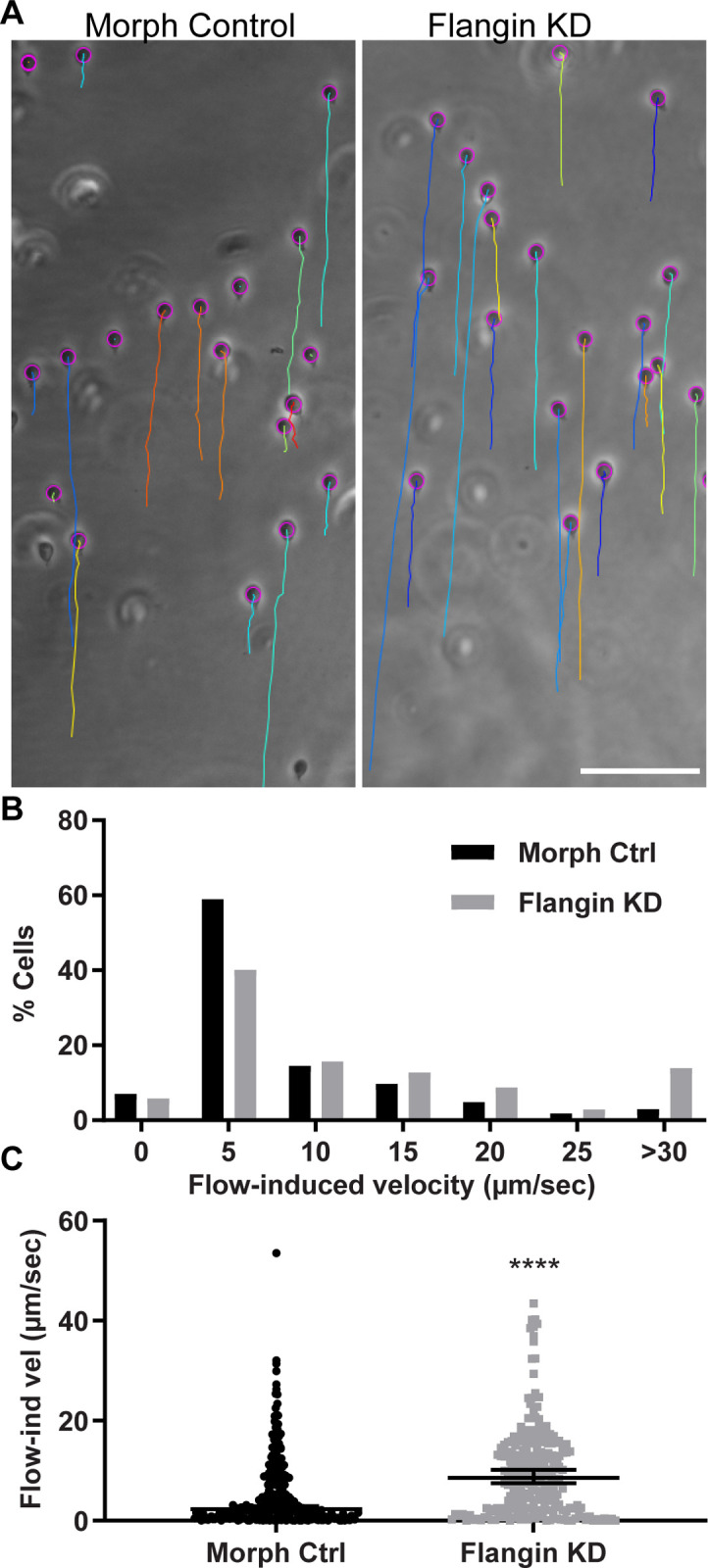
The flange has a role in attachment. (A) Flow chamber assay to test the role of the flange in attachment. A flow rate of 100μl/s induced sliding. The paths of cells were followed with TrackMate, the paths at the end of the 10s challenge are indicated by the colored lines. See S9 Movie. (B) Histogram of flow induced velocities for the standard morpholino control n = 268 and anti-Flangin morpholino treated cells n = 248 analyzed from six challenge assays. (C) Plot of measured values where error bars are median with 95% confidence interval; statistical significance determined with a Mann-Whitney U test. ****, P<0.0001. Scale bar = 100 μm.

To determine if the ventrolateral flange contributes to attachment, we used flow chambers to challenge morpholino control and Flangin-knockdown cells with fluid shear force (Fig 7A and S9 Movie). In this assay, Flangin-depleted cells were more frequently displaced and moved at higher flow induced velocities than control morpholino treated cells (Fig 7B and 7C). This result is consistent with the ventrolateral flange being an auxiliary mediator of attachment.

## Discussion

### Flangin and *Gl*Actin are structural components of the ventrolateral flange

We identified Flangin, a protein with three putative WH2-like domains and a Bro1 domain which are implicated in mediating interaction with actin. While *in vitro* assays testing direct interactions with actin are routine in model eukaryotes, it has proven challenging to produce sufficient quantities of *Gl*Actin to be used for such assays [14,17]. Therefore, we cannot yet conclude whether the WH2-like and/or Bro1 domains of Flangin directly mediate actin interaction. Yet, immunoprecipitation of HA-Flangin (Fig 1A) and the localization of Flangin to the tips of actin filaments (S3B Fig) indicate association with *Gl*Actin.

The reduction in flange width observed in *Gl*Actin- and Flangin-depleted cells indicates that these proteins are integral to the formation and maintenance of the ventrolateral flange. Live imaging revealed that Flangin is recruited to the ventrolateral flange concomitantly with flange formation after cell division (Fig 1C and S1 Movie). FRAP studies indicate that once in place, Flangin is part of a stable structure (Fig 3), which is consistent with the lack of dynamic growing and shrinking of the ventrolateral flange. In *Gl*Actin knockdown cells the collapsed flange likely reflects a stronger phenotype and more clearly highlights the critical role actin plays in driving flange protrusion (Fig 3A and 3B). Live imaging revealed that the flange protrudes from newly divided cells at a rate of around 0.4 μm/min until it reaches about 1 μm in width. Although *Giardia* is missing actin cytoskeleton proteins involved in cell motility, the protrusion rate is on the same order of magnitude as cells using lamellipodia dependent mesenchymal motility [41,42], suggesting that the ability of *Gl*Actin to establish membrane protrusions is intact. Indeed recent observations unexpectedly demonstrated that *Giardia* may be capable of phagocytosis, which in model eukaryotes is an actin-dependent process that requires the formation of large membrane protrusions [55].

Like *Gl*Actin depletion, Flangin-depletion resulted in reduced flange width, but during mitosis the flange can extend beyond the position of Flangin (Fig 2C). We propose that *Gl*Actin is necessary for flange membrane expansion, whereas Flangin is required for persistence of the protrusion. Tropomyosin, a coiled-coil protein that winds around and stabilizes actin filaments, has been shown to enhance the persistence of lamellipodial protrusions in fibroblasts [22]. By analogy, Flangin may have a role in stabilizing *Gl*Actin by either binding along its length or capping filaments. Co-localization of *Gl*Actin and Flangin suggests that Flangin is associated with actin filaments (Figs 1F, 2C and 5C). Re-sliced image stacks revealed that Flangin associates with the ends of filaments (S3B Fig). The development of a live *Gl*Actin marker or *in vitro* polymerization assays will be necessary to formally test how Flangin impacts *Gl*Actin dynamics.

### *Gl*Rac signaling is critical for ventrolateral flange assembly

Rho family GTPases regulate lamellipodia formation through coordination of the actin cytoskeleton [56,57]. We found *Gl*Rac and its corresponding signaling biosensor CRIB-mNG were both recruited to the flange (Fig 5A and 5B). *Gl*Rac depletion resulted in a serrated flange phenotype (Fig 5C–5E). These findings indicate that *Gl*Rac is an essential upstream regulator of flange formation and parallels the role of Rho family GTPases regulating actin and membrane protrusions in eukaryotes with conventional actin binding proteins [58]. That *Gl*Actin and *Gl*Rac are necessary for flange formation raises the interesting possibility that the flange has a shared origin with lamellipodia. Whether the flange’s resemblance to lamellipodia stems from a common origin or convergent evolution remains an intriguing question.

### The ventrolateral flange may have two critical roles in *Giardia*

In contrast to dynamic lamellipodia that drive cell motility, the flange is only dynamic during initial growth and then again during mitosis and cytokinesis. Our functional assays indicate that the flange strengthens trophozoite attachment (Fig 7A–7C). The increase in flange width during mitosis is a remarkable innovation. After the initiation of cytokinesis, the ventral adhesive disc is disassembled to redirect components to the nascent daughter ventral discs and also to clear a path for furrow progression (S3 Fig) [15,39]. Trophozoites remain attached until the initiation of cytokinesis, after which the trophozoites detach and swim apart to generate membrane tension for furrow progression and abscission [15,39]. To our knowledge, no other eukaryote can complete cytokinesis as quickly as *Giardia*. The incredibly fast cytokinesis is likely the result of evolutionary pressure to minimize the amount of time cells spend detached, which avoids being swept down the intestinal tract. We propose that the increased size of the flange during mitosis serves to maintain attachment when the parental ventral disc is being disassembled and expected to lack full function. On smooth surfaces the flange could contribute to attachment by maintaining suction under the cell, but the flange could also have adhesive properties as suggested by Erlandsen [28]. TIRF microscopy revealed that the ventrolateral flange accounts for a large portion of parasite surface contact. Adhesion could be mediated by plasma membrane localized lectins and alpha-giardins. Several studies have suggested that lectins contribute to *Giardia* attachment [12,59,60]. Recombinant alpha-1 giardin binds to human intestinal epithelial cells and alpha-1 giardin is enriched in the lateral crest and ventrolateral flange where it possibly contributes to adhesion [61]. Moreover, the ventrolateral flange has been observed in parasite-parasite interactions that suggest *Giardia* might also employ a cooperative mechanism of attachment to resist intestinal peristalsis [62]. The presence of redundant attachment mechanisms highlights the importance of intestinal attachment to the *Giardia* lifecycle.

The ventral disc and ventrolateral flange potentially contribute to attachment by creating chambers for holding a negative pressure differential under the cell. During the initial stage of attachment when cells are skimming the surface the flange and lateral crest form a C-shaped chamber where ventral flagella beating expels liquid to generate a negative pressure differential. By Stage IV of attachment when the bare area of the ventral disc has touched down, indicating full attachment, a requirement for flagella beating to maintain attachment is disputed [27,35]. Our TIRF microscopy observations indicate that Flangin and the ventrolateral flange are used to form a second chamber, the ventral groove, where flagella beating is hypothesized to establish negative pressures under the cell that is in addition to suction under the ventral disc. Indeed this secondary chamber is necessary to account for all the biophysical forces contributing to *Giardia* attachment [35,36], and our flow chamber experiments with Flangin knockdown cells indicate that the flange plays an important role in maintaining attachment.

An intriguing potential second role for the flange is to serve as a membrane reservoir to support cytokinesis. *Giardia* trophozoites are amongst the fastest dividing eukaryotes, requiring a median time of just 50 seconds to complete cytokinesis [15]. This timing does not include breaking of the fragile cytoplasmic bridge that sometimes persists from seconds to tens of minutes [39]. Division has a concomitant need for increased plasma membrane; for a typical eukaryotic cell, this involves an approximately 1.5 fold increase in surface area [63]. We previously proposed that *Giardia* might possess a reservoir of membrane to help support this rapid division [15]. In model eukaryotes, membrane protrusions have been shown to act as surface membrane reservoirs to support cell division [64,65]. The expanded mitotic flange could provide a large pool of surface membrane to support rapid furrow ingression. The observation that the flange is resorbed during cytokinesis suggests that flange membrane is needed for cytokinesis to proceed. Supporting this idea, the dominant negative N-terminal Bro1 domain that stabilizes the flange results in cytokinesis defects. These cells initiated cytokinesis normally but then furrow progression stalled or completely stopped presumably because membrane sequestered in the stabilized regions of the flange was needed for furrow progression and cytokinesis (Fig 4 and S7 Movie).

In summary, we identified Flangin, a protein that associates with *Gl*Actin and both proteins are critical for flange function. In parallel to the role of Rho GTPases regulating lamellipodia formation, *Gl*Rac regulates flange formation. In the future, we will work to identify the molecular effectors that recruit *Gl*Actin and Flangin to the flange, which is dynamically regulated over the cell cycle. Live imaging revealed that the flange grows to around 1 μm in width after cell division then remains static in interphase, grows during mitosis, and is resorbed during cytokinesis. We experimentally confirmed that the flange contributes to attachment and propose that this function is particularly important during mitosis when the flange is at its maximal width and the ventral disc is sequentially disassembled [15,39]. We also propose that flange has an innovative secondary role as a membrane reservoir to support *Giardia’s* rapid cytokinesis. Together, these observations point toward the biological importance of *Giardia* trophozoites minimizing time detached for their parasitic lifecycle. Therapeutic strategies targeting attachment would therefore effectively clear infection. The results presented here in combination with our recent study of *Gl*Actin’s role in ventral disc function indicate that *Giardia’s* highly divergent actin homolog has multiple roles in supporting attachment [5,6]. Inhibiting *Gl*Actin would simultaneously disrupt ventral disc and ventrolateral flange function, making it a promising therapeutic target.

## Materials and methods

### Pattern-Hit Initiated (PHI) BLAST for WH2 domain containing proteins

The *G*. *lamblia* genome was searched at NCBI with blastp using the PHI BLAST option with PHI pattern and input sequence combinations indicated in S1 Table.

### Strain and culture conditions

*Giardia* strain WB Clone 6 (ATCC 50803) was cultured as in [66].

### Vector construction

All constructs used in this study were made using standard techniques, see S2 Table for sequences and workflow. Construction of the morpholino sensitive HA-*Gl*Rac is described in reference [67].

### Immunoprecipitation

500 mL of *Giardia* wildtype and Flangin-3HA cell cultures were grown for 3 days, then iced for 2 hours to detach and pelleted at 1500xg at 4°C. Cells were then washed twice in HBS with 2XHALT protease inhibitors plus 10 μM chymostatin, 1μM leupeptin, and 1 μM E64. Each pellet was resuspended to a final volume of 1.2 mL. Then 100 mM DSP (dithiobis(succinimidyl propionate)) in DMSO was added to a final concentration of 1 mM and incubated at room temperature for 30 minutes. The reaction was quenched for 15 minutes with 20 mM Tris pH 7.4. Cells were then pelleted by spinning for 7 minutes at 700xg and resuspended in 350 μL lysis buffer (80 mM KCl, 10 mM imidazole, 1 mM MgCl2, 1 mM EGTA, 5% Glycerol, 20 mM HEPES, 0.2 mM CaCl2, 10 mM ATP, 0.1% Triton X-100, 250 mM NaCl, pH 7.2). Cells were lysed by sonication and cleared with a 10 minute spin at 10,000xg. A volume of 17.5 μL of equilibrated EZview Red Anti-HA Affinity gel (Sigma) was added to each tube of lysate, then incubated at 4°C with end-over-end mixing for 1 hour. Beads were then spun at 8,200xg for 30 seconds and the supernatant was discarded, followed by a total of three washes with 750 μL of lysis buffer. Each wash consisted of end-over-end rotation for 5 minutes followed by a 30 second spin at 8,200xg. The beads were then incubated with 50 μL of 8 M Urea at RT for 20 minutes to elute bound proteins without releasing heavy or light chains from the anti-HA antibody. Sample buffer was then added to eluted samples, boiled for 5 minutes, then run on 12% SDS-PAGE gel followed by Western blotting.

### Western blotting

Multi-plexed Western blots were performed by transferring proteins to Imobilon-FL membrane in Running Buffer (0.025M Tris, 0.192 M Glycine with 20% methanol, the blot was blocked in TBS (20mM Tris pH 7.6, 143 mM NaCl) plus 0.2% Tween-20 and 5% nonfat dry milk. Primary antibodies (anti-*Gl*Actin [14], anti-acetylated tubulin 6-11-B1 (Sigma), or anti-HA HA7 (Sigma)) were incubated overnight at 1:3000 at 4°C. After three washes (5, 10, 15 minutes) the blot was incubated for 60 minutes with secondary goat anti-rabbit-HRP (Bio-Rad, 1:7000) and Alexa 647 goat-anti-mouse antibodies (Molecular Probes, 1:2500). After three washes with TBS-T the blot was imaged with a Chemidoc MP (Bio-Rad). Values were normalized against the indicated loading controls after measuring band intensity with the ImageJ multi-measure function.

### Morpholino knockdown

Knockdown experiments were performed as described in [68] using the actin morpholino oligonucleotide 5’ GCAGGGTTGTCGTCTGTCATTTAC 3’, Flangin morpholino oligonucleotide 5’CGAGAGCGCAAGGATTCTGATGCAT 3’, *Gl*Rac morpholino oligonucleotide 5’ TATCCTCATTTCCTGTACTAGTCAT 3’, and the standard control morpholino oligonucleotide 5’ CCTCTTACCTCAGTTACAATTTATA 3’, sourced from Gene Tools LLC. Analysis was performed approximately 24 hours after morpholino treatment.

### Immunofluorescence and expansion microscopy

*Gl*Actin localization at the flange was not apparent in our prior localization studies, fixation in culture tubes does not preserve display of the flange after settling in random orientations on cover glass. To observe the flange, we allowed live cells in growth medium to attach to poly-L-lysine coated cover glass before fixation. Fixed imaging was modified from [67] by including the starve and release (3.5 hours) method to increase mitotic index [66]. For expansion microscopy fixation and staining proceeded as in [15]. The expansion steps were performed as described in [34,69]. Briefly, detached cells were allowed adhere to poly-L-lysine coated 12 mm round coverglass for 1h in an atmosphere-controlled incubator containing 5% C0_2_, 2% O_2_, 93% N_2_ at 37°C. Samples were immediately fixed with warm 3.2% PFA in PEM buffer (100 mM PIPES, 1 mM EGTA, 1 mM MgCl_2_, pH 6.9) for 20 min at 37°C, followed by gentle washing with PBS. For immunostaining, samples were incubated overnight at 4°C with anti-HA antibody (Roche 11867423001, 2.5 μg/mL), followed by one hour each of Mouse Anti-Rat antibody and Donkey Anti-Mouse antibody (5 μg/mL) that had been modified with Alexa Fluor 488 to have ~5 dyes/protein. All immunostaining steps were conducted in PEMBALG (50 mM PIPES, 5 mM EGTA, 10 mM MgSO_4_, 1% BSA, 0.1 M lysine, 0.5% gelatin, 3 mM NaN_3_, and 0.1% TX100) and each step was followed by three PBS exchanges for at least 15 min. After immunostaining, the sample were exposed to a 25 mM solution of methacrylic acid N-hydroxysuccinimide ester in PBS for 30 min, washed in PBS, then incubated in monomer solution (8.6% sodium acrylate, 20% acrylamide, 0.075% bis-acrylamide, 2 M NaCl, 1× PBS) for 5 min. After removing excess monomer, the sample was inverted onto a 70 μL droplet of freshly prepared monomer solution containing 0.2% (w/w) of both APS and TEMED and polymerized for 20 min. After polymerization, the sample was digested overnight in digestion buffer (0.8 M guanidine hydrochloride, 1× TAE, 0.5% TX100, pH 8.3) containing ∼6 U/mL of Proteinase K at 37°C. Finally, the sample was expanded for ∼2 h in an excess volume of deionized water, with water exchanges every ∼30 min until complete. Detailed protocols including gel handling are available in references [34,69]. Images were collected on a Leica SP5 II using a 63× water objective, NA 1.2 PMT at 700 volts, Pinhole size 1 airy unit, 488 nm laser at 20% power.

### Fluorescent live imaging

Cells were chilled on ice for 20 minutes to detach from the culture tube and then placed into an Attofluor cell chamber (Molecular Probes) and incubated in a GasPak EZ anaerobic pouch (BD) or a Tri-gas incubator (Panasonic) set to 2.5% O_2_, 5% Co_2_ for 1–2 hours at 37° C. Cells were then washed four times with SB5050 (0.1% K2HP04, 0.06% KH2P04, 1% glucose, 0.2% NaCl, 0.2% cysteine-HCI monohydrate, 0.02% ascorbic acid, 0.0228% ferric ammonium citrate, 0.05% bovine bile and 5% bovine serum, pH 7.1). Cells were overlaid with a mixture of 0.7–1% ultra-low gelling agarose (Sigma A2576) melted in HBS (137mM NaCl, 5mM KCl, 0.91mM Na_2_HpO_4_-heptahydrate, 5.55mM Glucose, 20mM HEPES, and pH7) and diluted into SB5050, left at room temperature for 10 minutes to solidify the agarose. Imaging was performed under 2.5% O_2_, 5% CO_2_, and 37° C (Oko Lab Boldline CO_2_/O_2_). For imaging of mitotic cells, the cells were treated with 0.25μM Albendazole ~4 hours to increase the mitotic index. Albendazole was washed out with fresh medium to release the cell cycle block before imaging. For Halo-tag labeling cells were allowed to re-attach for 1 hour under hypoxic conditions and then incubated with 0.5 μM of Janelia Fluor 549 (Promega) for 15 minutes. Cells were then washed four times with pre-warmed growth medium and then allowed to incubate another 30 minutes in growth medium to allow diffusion of unbound intracellular dye out of the cell before proceeding to image as above. Time-lapse imaging was performed on a DeltaVision Elite deconvolution microscope (GE, Issaquah, WA) equipped with DIC optics, using a 100x 1.4 NA or 60x 1.42 NA objective, and a PCO Edge 5.4 sCMOS camera (PCO-TECH Inc).

### Ventrolateral flange width measurements

Unless otherwise indicated ventrolateral flange measurements were taken from DIC images at the anterior apex of the cell as shown in Fig 2A using the ImageJ multi-measure function. This region was chosen because it is where flange growth is initiated after cytokinesis and the cell body does not obscure measurements here. Note that the cell body does obscure viewing the base of the flange on the left and right sides of the cell with DIC optics (Fig 1).

### Fluorescence recovery after photobleaching

Attofluor cell chambers were seeded with Flangin-mNG parasites as outlined above. Imaging was conducted using a FRAP-enabled DeltaVision Spectris, 488 nm solid-state laser, 100% laser power, 50 mW laser, and 25 ms stationary pulse. After acquiring a pre-bleach image the first post bleach image was acquired approximately 2 ms after the bleach event. During the first minute, images were acquired every 10 second for 1 minute, then every 20 seconds for the following 2 minutes, every 50 seconds over the following 6 minutes, and finally, every 100 seconds for the remainder of the experiment. Normalized mNG fluorescence recovery was calculated by subtracting the background noise from the ROI intensity measurement; the background-subtracted intensity measurement was then divided by a fluorescent control ROI intensity measurement to normalize for photobleaching due to imaging.

### Scanning electron microscopy

Performed as described in [39]. Briefly, 3% glutaraldehyde in 0.1M cacodylate buffer was used for trophozoite fixation, and 1% OsO_4_ in 0.1M cacodylate buffer for postfixation. Washing steps included 5% sucrose in cacodylate buffer, and PBS. Dehydration proceeded in an ethanol series prior to critical point drying and gold coating. Imaging was performed on a JEOL 6300 scanning electron microscope.

### Focused Ion beam-scanning electron microscope tomography

Trophozoites were allowed to attach to laser marked slides (LASERMarking, Munich, Germany) in a silicon anaerobic chamber at 37°C. Slides were rinsed with PBS, pH 7.2 and immediately fixed with 2.5% (v/v) glutaraldehyde (Science Services GmbH, Munich, Germany) in 75 mM cacodylate (Sigma-Aldrich), 75 mM NaCl, 2 mM MgCl 2 for 30 min, followed by 3 washing steps in cacodylate buffer. Cells were stained with DAPI, sealed with a coverslip and Fixogum (Marabu GmbH, Tamm, Germany) to prevent drying during LM investigation on Zeiss Axiophot fluorescence light microscope. The positions of cells of interest (ROIs) were marked on a template, with the same coordinates. For documentation, epifluorescence, phase contrast and DIC images were taken to retrieve the ROIs in FIB/SEM. All other steps, i.e. postfixation, ultrathin embedding, mounting, imaging in a Zeiss Auriga FIB/SEM workstation operating under SmartSEM (Carl Zeiss Microscopy, Oberkochen, Germany) and image analysis using Amira (Thermo Fisher Scientific, USA) were carried out as described in [70,71].

### Total Internal Reflection (TIRF) microscopy

After seeding cells in AttoFluor chambers as described above, CellMask Deep Red Plasma membrane Stain (ThermoFisher) was used in according to manufacturer’s instructions after medium replacement with 1X HBS. Cells were imaged at 37°C in a chamber with 2% CO2 and 5% O2 with a DeltaVision OMX (GE) using 60x TIRF objective. Images were taken from randomly selected fields of view. Data is from three independent experiments.

### Cell attachment assays

Cells were depleted for either Flangin, *Gl*Actin, or *Gl*Rac (see above for morpholino protocol), allowed to recover for 24 hours in a total volume of 13 mL of medium before performing the attachment assays.

Basic Assay: Culture tubes were inverted several times and then the detached cells were decanted and counted with a MoxiZ (Orflo) coulter counter. The medium was replaced with 13 mL of ice-cold medium and then placed on ice for 30 minutes to detach the remaining attached cells. These cells were then counted as above. Three independent replicates of each cell line and control were analyzed.

Flow Chamber Assay: Microfluidic devices were fabricated using a silicon master, which was created using deep reactive ion etching. The silicon master contained a raised channel that had a rectangular cross-sectional area, with a length of 1 cm, a width of 1 mm, and a height of 115 μm. A polydimethylsiloxane (PDMS) (Sylgard 184, Dow Corning) mold was made using a 10:1 base to curing agent ratio, which was mixed for 5 minutes, degassed for 20 minutes, poured over the master, and placed in an oven for 10 minutes at 110°C to cure. The mold was peeled away from the silicon master and holes were punched for the inlet and outlet of the channel. Afterwards, inlet and outlet ports were created using a custom aluminum mold with pegs to insert the silicone tubing (0.040 inch ID/ 0.085 inch OD, HelixMark). Degassed 10:1 PDMS was poured into the preheated aluminum mold and cured at 110°C for 1 hour. The inlet and outlet ports were aligned with holes on the top face of the microfluidic device and stuck together using uncured PDMS and 5 minutes of oven heat. Finally, the bottom face of the microfluidic device and a clean glass slide were plasma treated for 10 seconds and aligned together to create a water-tight seal between the two layers. Trophozoites were chilled for 20 minutes on ice and then loaded into microfluidic devices placed in a GasPak EZ anaerobic pouch (BD Scientific) at 37 °C for 1 hour to allow re-attachment. Warm growth medium was drawn into a disposable syringe (BD Scientific) and then loaded onto a syringe pump (Harvard Apparatus). For experiments, the flow rate was set to 20 μL/s for 30 seconds to clear swimming trophozoites and then ramped to 100 μL/s over 5 seconds and challenged for 60 seconds. The challenge assays were recorded at 2 FPS using a Nikon Eclipse Ti microscope with a 20× phase objective, Clara DR-1357 CCD camera (Andor), and a live-cell incubation chamber (In Vivo Scientific, Inc) to maintain a temperature of 37 °C. Trophozoite displacement was quantified starting at 35 seconds using the TrakMate ImageJ plugin via manual tracking over the first 10 seconds of 100 μL/s flow [72,73].

### Statistical analysis

Statistically significant differences between knockdown and control groups were obtained using GraphPad Prism 7.02 software and unpaired t test with Welch’s correction for parametric data and the Mann-Whitney U test for non-parametric data.

## Supporting information

S1 Fig Variations in the amino acid sequence of *Gl*Actin are minimal within the WH2 binding region. Modeled surface of *Gl*Actin based on mammalian skeletal actin (PDB 2A3Z). Grey surface shows conserved residues, with red sections highlighting variations between skeletal actin and *Gl*Actin. Pink resides are those residues that differ and are within 5 Å of the bound WH2 domain of WASP (blue ribbon). *Inset*: Only one reside change, Ser350Ala, would cause the loss of a hydrogen bond between the WH2 domain and *Gl*Actin (ribbons with WH2 residue Gln437 and Ser350 shown as sticks). Residue 437 is not part of the conserved WH2 motif [20].
(TIF)

S2 Fig Diagram of Flangin domain organization. (A) PHI BLAST with DM SCAR AAF53042 as the query sequence identified three WH2-like domains in Flangin (GL50803_7031). (B) Diagram of Flangin domain organization with amino acid positions indicated.
(TIF)

S3 Fig Full size IP Western blots. Anti-HA in red and anti-*Gl*Actin in green. Top blot shows the input and lower blot shows the output from the anti-HA beads.
(TIF)

S4 Fig The flange grows in width before ventral disc disassembly in telophase. Immunofluorescence localization of tubulin, *Gl*Actin (green), Flangin-HA (magenta), and DNA (blue), throughout the cell cycle. *Gl*Actin and Flangin localized to the flange during interphase and mitosis. The flange was wider in mitosis corresponding to when the ventral disc begins conformational changes and disassembly as indicated by the size of the bare area (Arrows). This microtubule free region grows as the ventral disc is disassembled in telophase. Ultimately the ventral disc opens up and completely disassembles to permit furrow progression during cytokinesis. The insets show magnified views of *Gl*Actin and Flangin in the flange. The flange is resorbed during cytokinesis, which corresponds to when the cells begin to swim apart to generate membrane tension. Note that the interphase and mitotic cells are partial projections optimized to show *Gl*Actin localization in the flange, while the entire Z-stack was projected for the cells in cytokinesis to show that the flange has been resorbed. Also see Fig 1 and S1 Movie of reference [15], which shows microtubule and flange dynamics in live cells. (B) Flangin (magenta) can be seen at the ends of *Gl*Actin filaments (green) in maximal projections and re-sliced image stacks. Scale bar = 5 μm.
(TIF)

S5 Fig Morpholino efficacy. (A) Standard control morpholino versus translation blocking anti-*Gl*Actin treatment specifically reduced *Gl*Actin by ~60% as observed by Western blotting. Protein levels were normalized using tubulin as a loading control. Note this is a verification of a previously published morpholino (B) Standard control morpholino versus translation blocking anti-Flangin morpholino reduced Flangin levels by 70.2% ± 7.7% based on Western Blotting (n = 6). Protein levels were normalized using actin as a loading control (C) Standard control morpholino versus translation blocking anti-*Gl*Rac morpholino reduced *Gl*Rac levels by ~70%. Protein levels were normalized using actin or tubulin as a loading control. Note this is a verification of a previously published morpholino. All blots were performed 24 h after morpholino treatment, matching the timing of live and fixed cell experiments.
(TIF)

S6 Fig Localization of the N-terminal Bro1 domain and C-terminal WH2 domain fragments in live cells. (A) Induction test of the N-terminal Bro1 fragment. Images are equal exposure and scaling. (B) The N-terminal Bro1 fragment is recruited to the same regions as full length Flangin. Expression of this fragment results in failed cytokinesis, asterisk marks cell actively trying to divide that failed to break down the flange. (C) Induction test of the C-terminal region containing putative WH2-like domains. Images are equal exposure and scaling. (D) The C-terminal WH2 domain containing fragment is not specifically recruited to the flange. Scale bar = 10 μm.
(TIF)

S7 Fig (A) Quantifying the role of Flangin, *Gl*Rac, and actin in non-challenged cell attachment. Each protein was depleted and then attached and unattached cells were counted. Three technical replicates for unattached cells were conducted. Mean ± SEM n = 3, control 13.73 ± 0.78, Flangin 14. 81 ± 0.62, *Gl*Rac 31.23 ± 0.83, and *Gl*Actin 54.48 ± 0.25. *Gl*Rac and *Gl*Actin had statistically significant reduction in their ability to attach versus the morpholino control, t-test ***, P< 0.001.
(TIF)

S1 Movie Flangin dynamics during mitosis and cytokinesis. Note that in a portion of cells a cytoplasmic bridge can persist between daughter cells for tens of minutes [39].
(AVI)

S2 Movie DIC imaging of cytokinesis and flange regrowth. Video also shows the flange remains size uniform after re-growth (filmed for >50 minutes).
(AVI)

S3 Movie Time lapse image of cell analyzed in Fig 3. Movement during entire time series indicates viability.
(AVI)

S4 Movie DIC imaging of morpholino control cell through mitosis and cytokinesis. (AVI)

S5 Movie DIC imaging of a Flangin depleted cell that restores flange width before cytokinesis. (AVI)

S6 Movie A dominant negative Flangin_1-362_-mNG cell that fails to retract the flange and fails cytokinesis. Note that this cell remains attached to the cover glass and the flange width increases as filopodia like projections are formed.
(AVI)

S7 Movie Dominant negative Flangin_1-362_-mNG imaged with DIC only to avoid oxidative stress. This cell completes cytokinesis at 8 minutes, the delay is associated with partial failure to resorb the flange.
(AVI)

S8 Movie Deployment of flange during attachment. As this cell attaches the flange can be seen to unfold and engage with the cover glass.
(AVI)

S9 Movie Representative attachment assay under flow. Morpholino control on the left and Flangin knockdown on the right. Colored lines indicate the trajectory of individual cells beginning at 35 seconds when the flow rate peaks at 100 μL/s.
(AVI)

S1 Table Spreadsheet containing putative WH2 domain containing proteins. (XLSX)

S2 Table Spreadsheet containing primer sequences and construct design. (XLSX)
