# Supplementary figures and images for: The Giardia ventrolateral flange is a lamellar membrane protrusion that supports attachment
Source: PLoS Pathog. 2022 Apr 28;18(4):e1010496. doi: 10.1371/journal.ppat.1010496 (PMC9089883; doi:10.1371/journal.ppat.1010496)

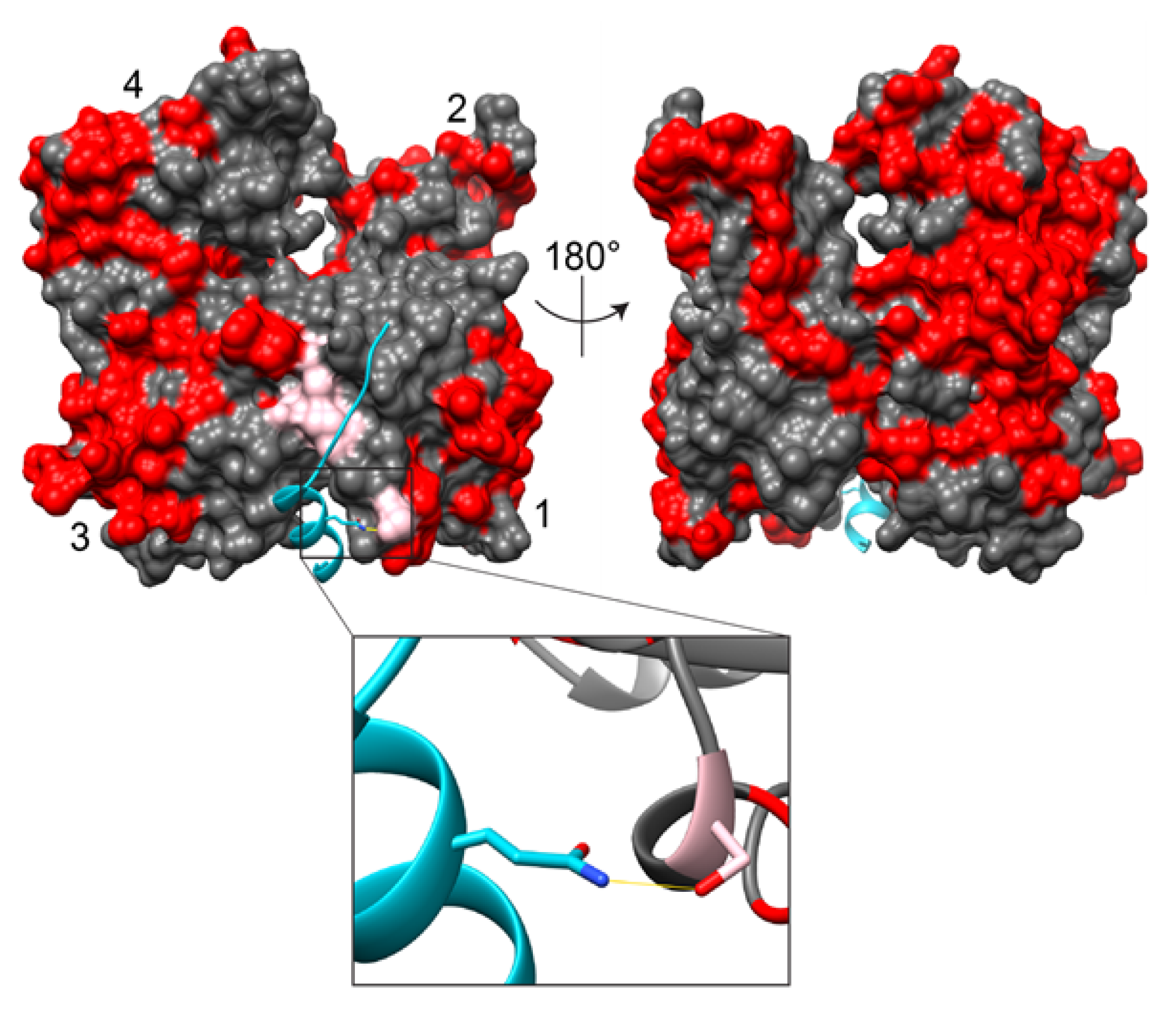

Supplement: S1 Fig — Modeled surface of GlActin based on mammalian skeletal actin (PDB 2A3Z). Grey surface shows conserved residues, with red sections highlighting variations between skeletal actin and GlActin. Pink resides are those residues that differ and are within 5 Å of the bound WH2 domain of WASP (blue ribbon). Inset: Only one reside change, Ser350Ala, would cause the loss of a hydrogen bond between the WH2 domain and GlActin (ribbons with WH2 residue Gln437 and Ser350 shown as sticks). Residue 437 is not part of the conserved WH2 motif [20]. (TIF) [file ppat.1010496.s001.tif]

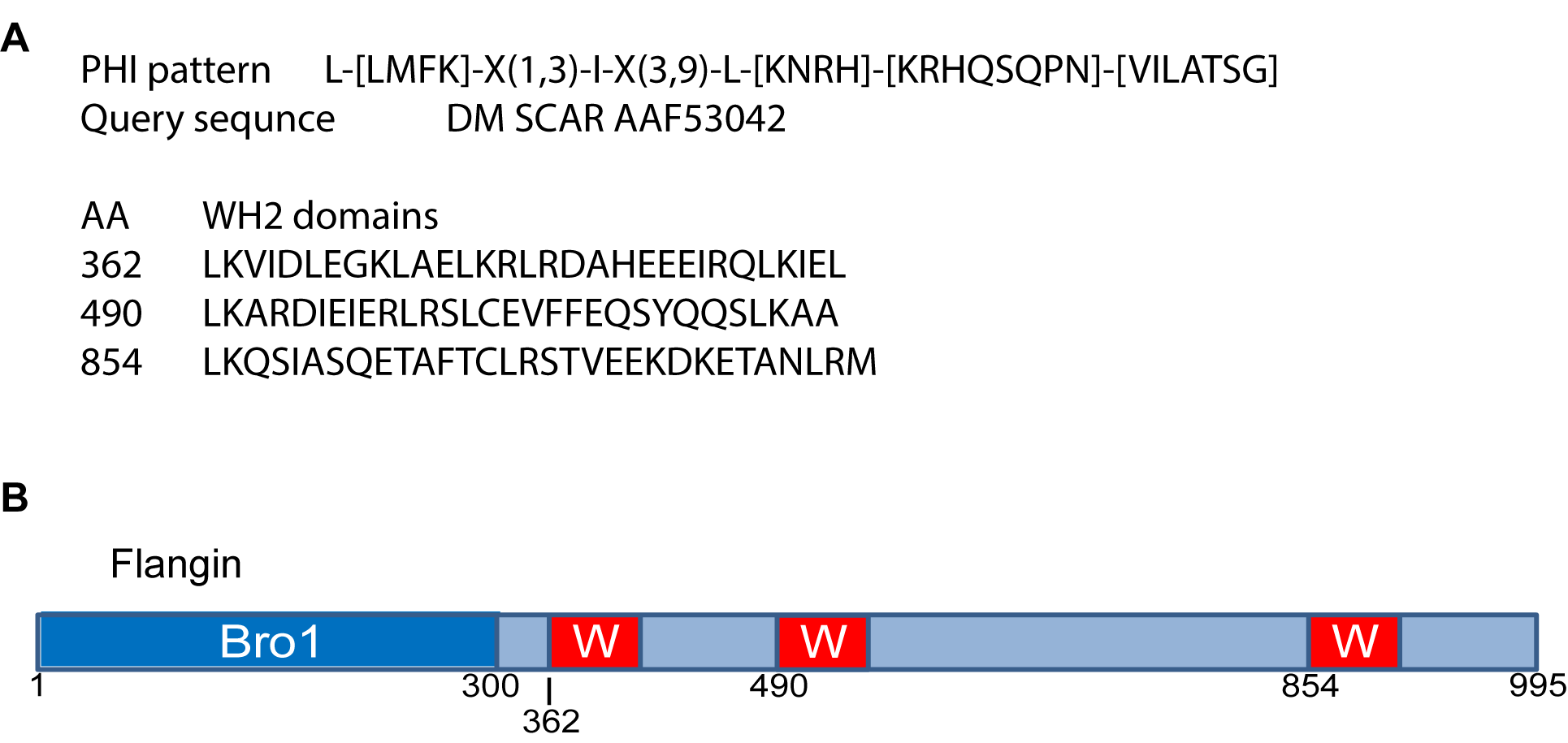

Supplement: S2 Fig — (A) PHI BLAST with DM SCAR AAF53042 as the query sequence identified three WH2-like domains in Flangin (GL50803_7031). (B) Diagram of Flangin domain organization with amino acid positions indicated. (TIF) [file ppat.1010496.s002.tif]

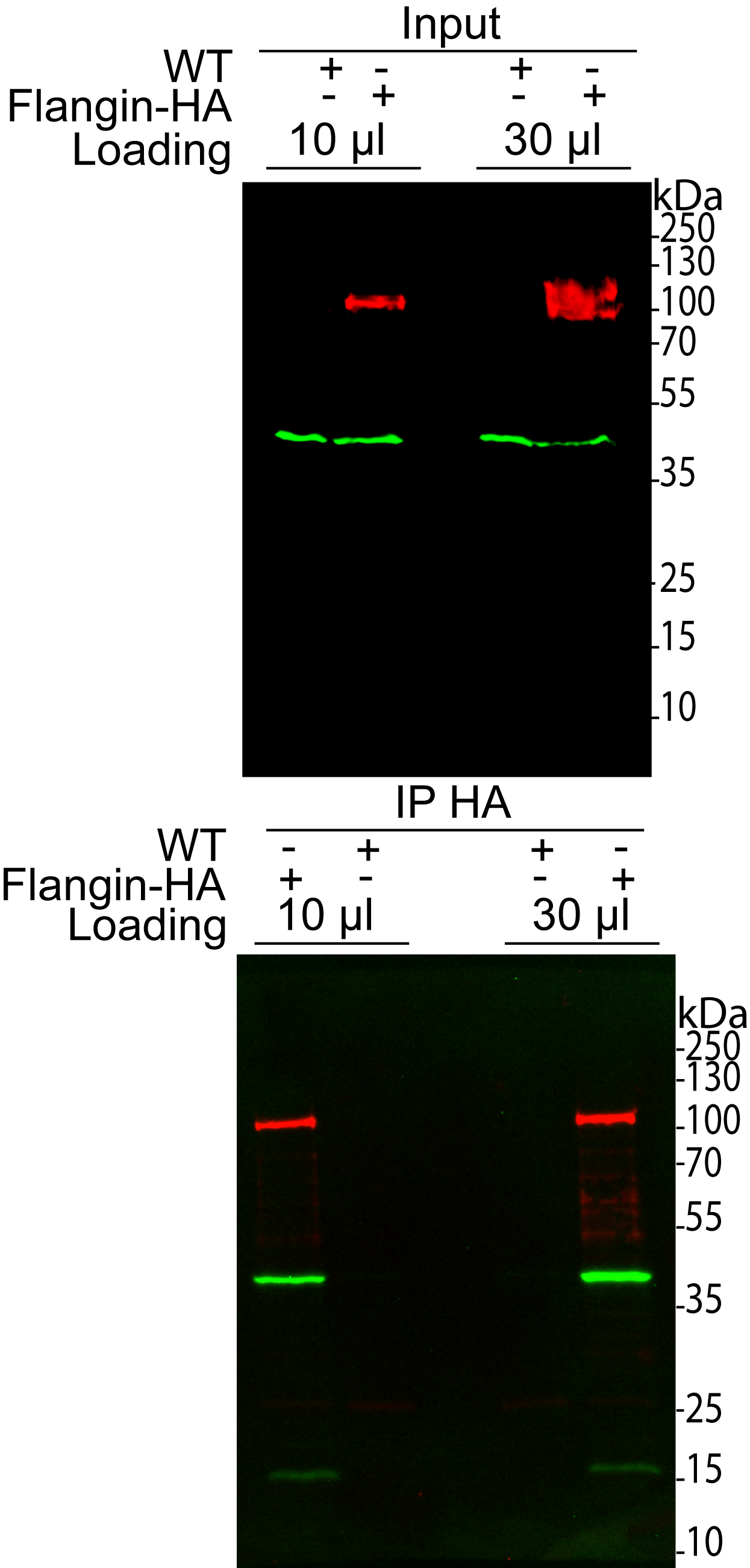

Supplement: S3 Fig — Anti-HA in red and anti-GlActin in green. Top blot shows the input and lower blot shows the output from the anti-HA beads. (TIF) [file ppat.1010496.s003.tif]

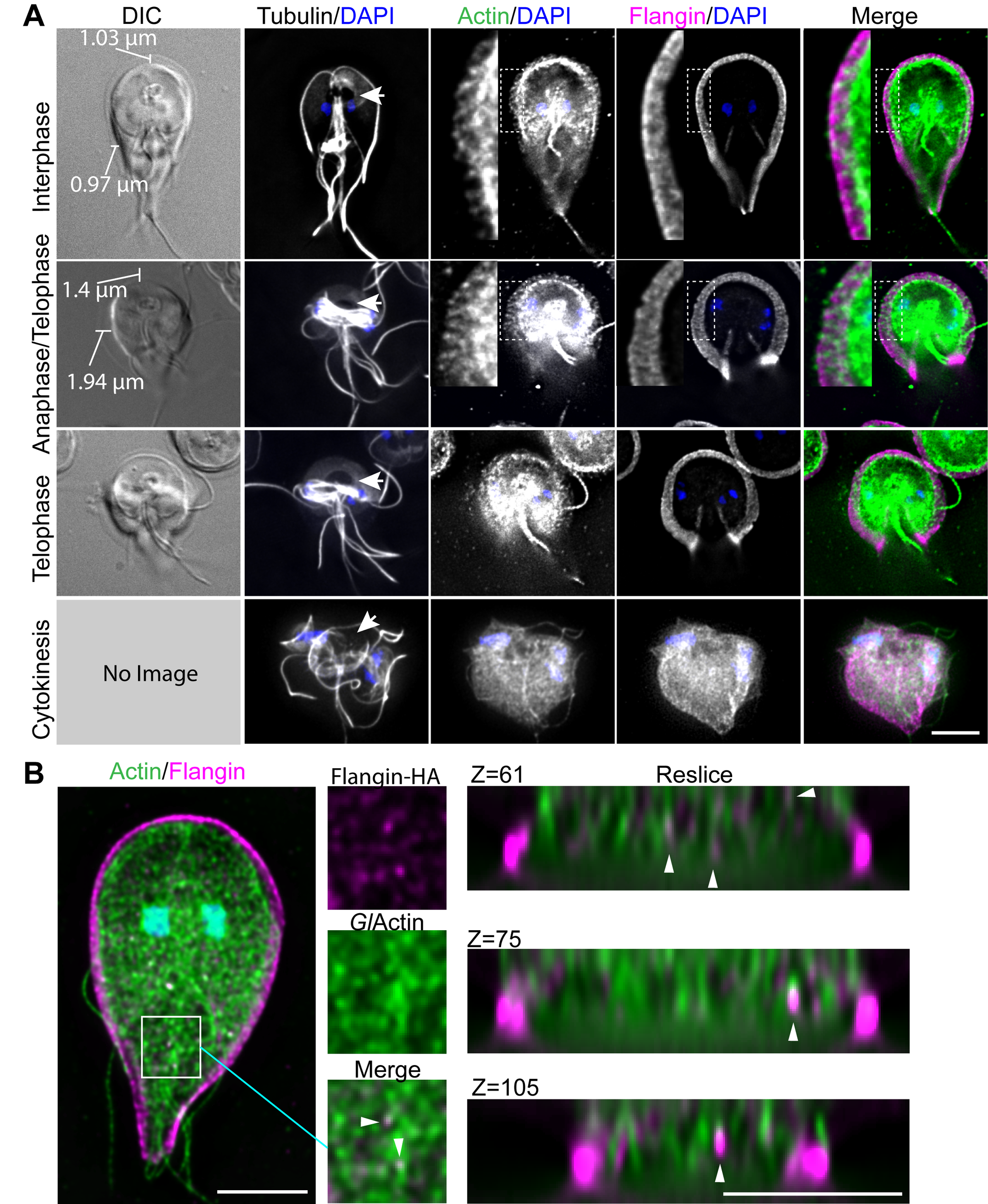

Supplement: S4 Fig — Immunofluorescence localization of tubulin, GlActin (green), Flangin-HA (magenta), and DNA (blue), throughout the cell cycle. GlActin and Flangin localized to the flange during interphase and mitosis. The flange was wider in mitosis corresponding to when the ventral disc begins conformational changes and disassembly as indicated by the size of the bare area (Arrows). This microtubule free region grows as the ventral disc is disassembled in telophase. Ultimately the ventral disc opens up and completely disassembles to permit furrow progression during cytokinesis. The insets show magnified views of GlActin and Flangin in the flange. The flange is resorbed during cytokinesis, which corresponds to when the cells begin to swim apart to generate membrane tension. Note that the interphase and mitotic cells are partial projections optimized to show GlActin localization in the flange, while the entire Z-stack was projected for the cells in cytokinesis to show that the flange has been resorbed. Also see Fig 1 and S1 Movie of reference [15], which shows microtubule and flange dynamics in live cells. (B) Flangin (magenta) can be seen at the ends of GlActin filaments (green) in maximal projections and re-sliced image stacks. Scale bar = 5 μm. (TIF) [file ppat.1010496.s004.tif]

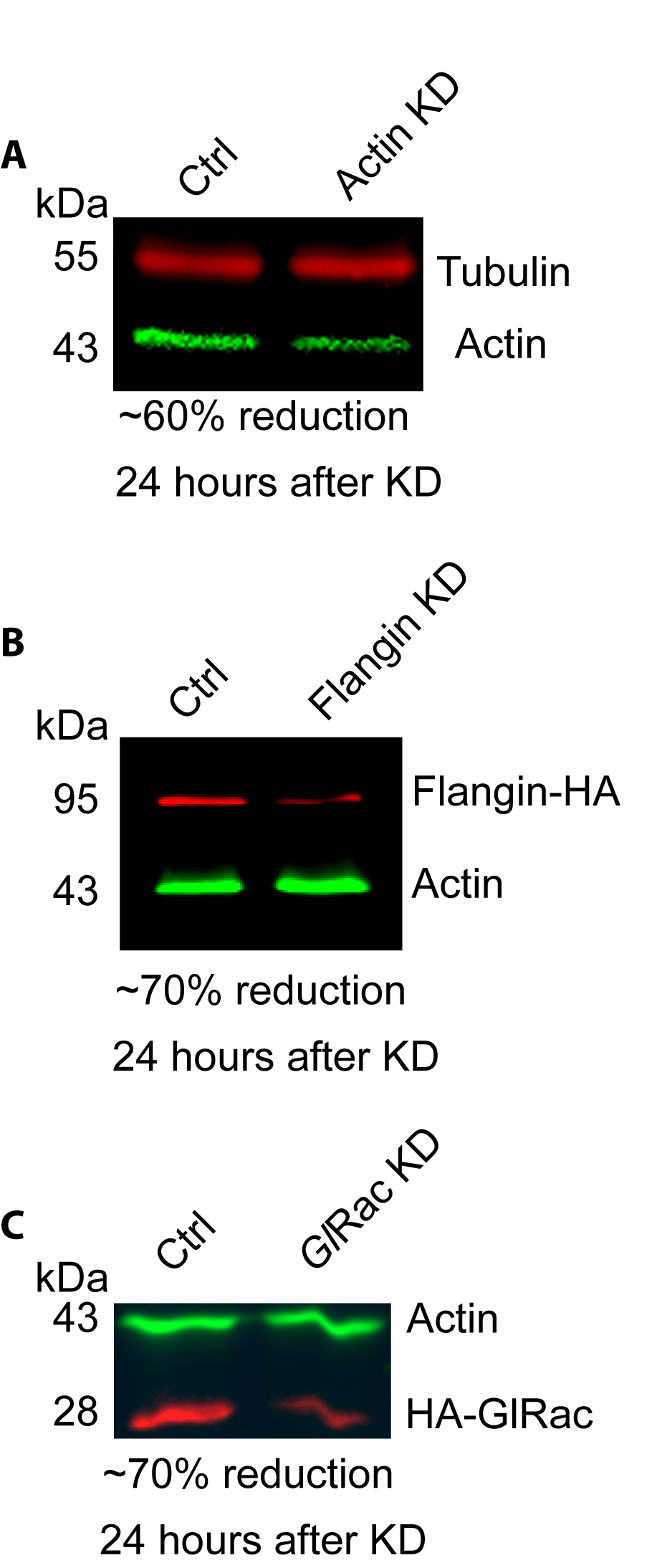

Supplement: S5 Fig — (A) Standard control morpholino versus translation blocking anti-GlActin treatment specifically reduced GlActin by ~60% as observed by Western blotting. Protein levels were normalized using tubulin as a loading control. Note this is a verification of a previously published morpholino (B) Standard control morpholino versus translation blocking anti-Flangin morpholino reduced Flangin levels by 70.2% ± 7.7% based on Western Blotting (n = 6). Protein levels were normalized using actin as a loading control (C) Standard control morpholino versus translation blocking anti-GlRac morpholino reduced GlRac levels by ~70%. Protein levels were normalized using actin or tubulin as a loading control. Note this is a verification of a previously published morpholino. All blots were performed 24 h after morpholino treatment, matching the timing of live and fixed cell experiments. (TIF) [file ppat.1010496.s005.tif]

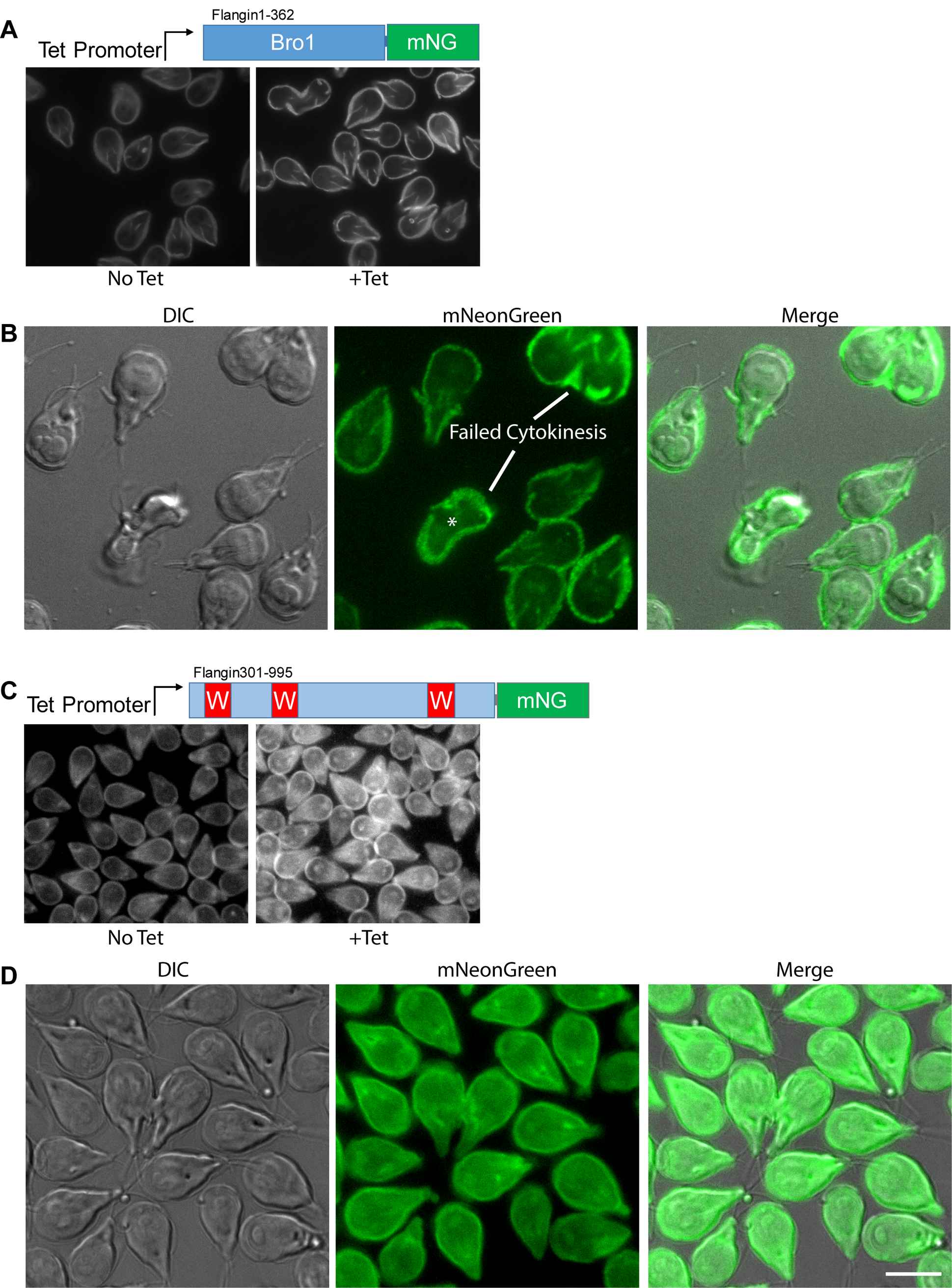

Supplement: S6 Fig — (A) Induction test of the N-terminal Bro1 fragment. Images are equal exposure and scaling. (B) The N-terminal Bro1 fragment is recruited to the same regions as full length Flangin. Expression of this fragment results in failed cytokinesis, asterisk marks cell actively trying to divide that failed to break down the flange. (C) Induction test of the C-terminal region containing putative WH2-like domains. Images are equal exposure and scaling. (D) The C-terminal WH2 domain containing fragment is not specifically recruited to the flange. Scale bar = 10 μm. (TIF) [file ppat.1010496.s006.tif]

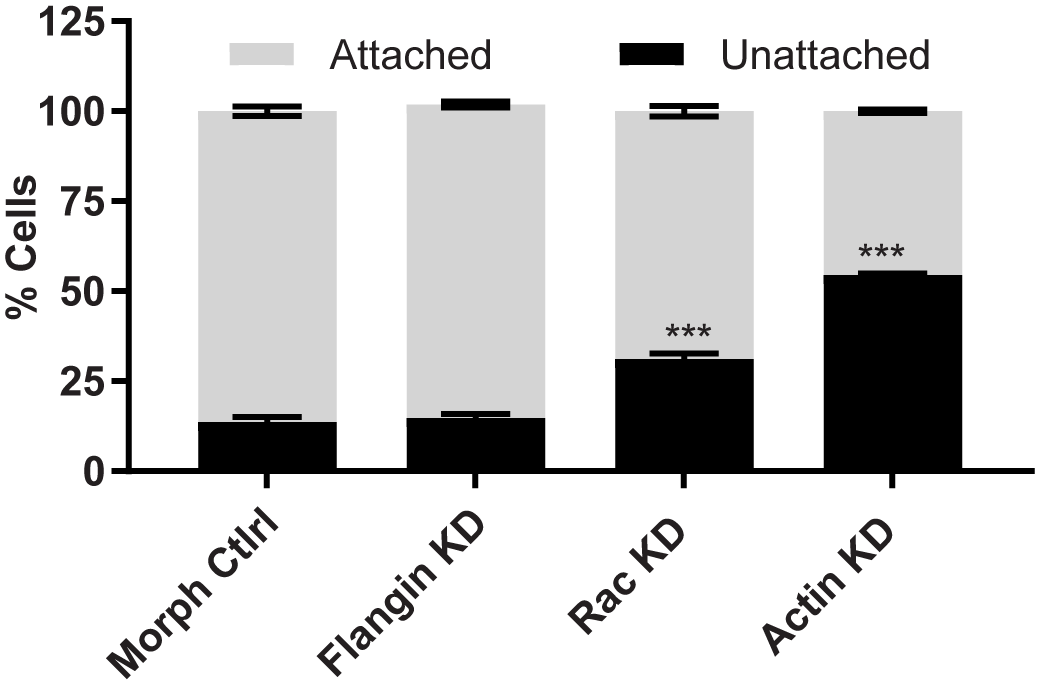

Supplement: S7 Fig — (A) Quantifying the role of Flangin, GlRac, and actin in non-challenged cell attachment. Each protein was depleted and then attached and unattached cells were counted. Three technical replicates for unattached cells were conducted. Mean ± SEM n = 3, control 13.73 ± 0.78, Flangin 14. 81 ± 0.62, GlRac 31.23 ± 0.83, and GlActin 54.48 ± 0.25. GlRac and GlActin had statistically significant reduction in their ability to attach versus the morpholino control, t-test ***, P< 0.001. (TIF) [file ppat.1010496.s007.tif]
